# Genome-wide analysis of condensin binding in *Caenorhabditis elegans*

**DOI:** 10.1186/gb-2013-14-10-r112

**Published:** 2013-10-14

**Authors:** Anna-Lena Kranz, Chen-Yu Jiao, Lara Heermans Winterkorn, Sarah Elizabeth Albritton, Maxwell Kramer, Sevinç Ercan

**Affiliations:** 1 Department of Biology, Center for Genomics and Systems Biology, New York University, New York, NY 10003, USA

## Abstract

**Background:**

Condensins are multi-subunit protein complexes that are essential for chromosome condensation during mitosis and meiosis, and play key roles in transcription regulation during interphase. Metazoans contain two condensins, I and II, which perform different functions and localize to different chromosomal regions. *Caenorhabditis elegans* contains a third condensin, I^DC^, that is targeted to and represses transcription of the X chromosome for dosage compensation.

**Results:**

To understand condensin binding and function, we performed ChIP-seq analysis of *C. elegans* condensins in mixed developmental stage embryos, which contain predominantly interphase nuclei. Condensins bind to a subset of active promoters, tRNA genes and putative enhancers. Expression analysis in *kle-2*-mutant larvae suggests that the primary effect of condensin II on transcription is repression. A DNA sequence motif, GCGC, is enriched at condensin II binding sites. A sequence extension of this core motif, AGGG, creates the condensin I^DC^ motif. In addition to differences in recruitment that result in X-enrichment of condensin I^DC^ and condensin II binding to all chromosomes, we provide evidence for a shared recruitment mechanism, as condensin I^DC^ recruiter SDC-2 also recruits condensin II to the condensin I^DC^ recruitment sites on the X. In addition, we found that condensin sites overlap extensively with the cohesin loader SCC-2, and that SDC-2 also recruits SCC-2 to the condensin I^DC^ recruitment sites.

**Conclusions:**

Our results provide the first genome-wide view of metazoan condensin II binding in interphase, define putative recruitment motifs, and illustrate shared loading mechanisms for condensin I^DC^ and condensin II.

## Background

**Figure 1:**
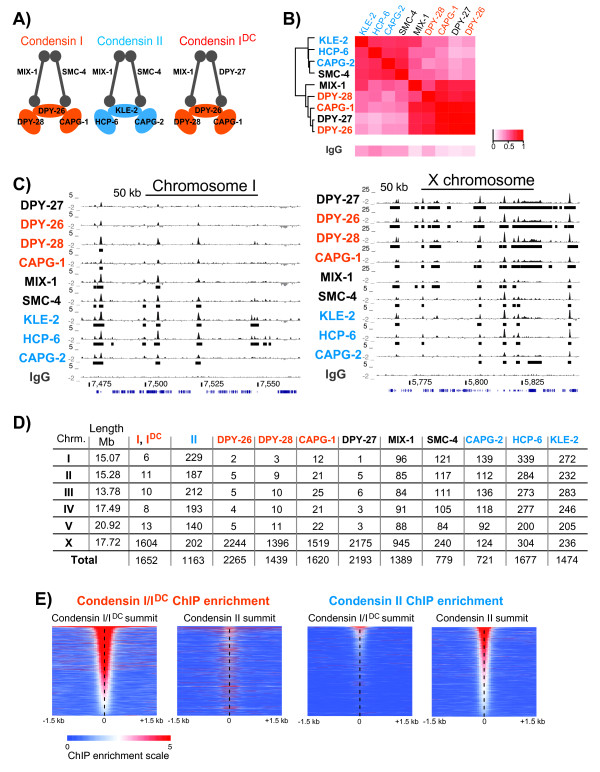
**Chromatin immunoprecipitation followed by high-throughput sequencing (ChIP-seq) patterns of the three condensins in *Caenorhabditis elegans *embryos. (A)** Subunit composition of the three *C. elegans* condensin complexes. Condensin I and II share SMC-4 and MIX-1, and are distinguished by three non-SMC subunits. DPY-27, an SMC protein, is the only subunit different between condensin I and the dosage compensation condensin I^DC^. Non-SMC subunits of condensin I-I^DC^ and condensin II are in orange and blue, respectively. **(B)** Median ChIP-seq enrichment in 1 kb contiguous windows across the genome were used to calculate pairwise Pearson correlation coefficients, clustered with hierarchical clustering, and plotted as a heat map. The subunits of each condensin type cluster closer together. **(C)** University of California Santa Cruz (UCSC) genome browser view of ChIP-seq enrichment scores of each condensin subunit across a 200 kb region on chromosome I (left) and X chromosome (right). Note that on the X chromosome condensin I-I^DC^ subunit (orange) ChIP-enrichment scores are much greater; the scale is five times larger than that of condensin II subunits (blue). **(D)** The number of ChIP-seq binding peaks per condensin subunit per chromosome is shown. Condensin I^DC^ mostly binds to the X chromosome, whereas condensin II is similarly distributed across all chromosomes. **(E)** Average ChIP-seq enrichment score from non-SMC subunits of condensin I-I^DC^ and II are plotted across the summit of each condensin I-I^DC^ or condensin II peak. The peaks are ordered by ChIP enrichment (highest ChIP value on top), to illustrate that condensin II binds to highest ChIP enrichment sites of condensin I-I^DC^. ChIP-seq, chromatin immunoprecipitation followed by high-throughput sequencing; SMC, structural maintenance of chromosomes.

Condensins are evolutionarily conserved protein complexes that function in a wide-range of cellular processes including chromosome condensation, segregation, transcription regulation and DNA repair [1-4]. Metazoans contain two types of condensins (I and II) that share a heterodimer of two structural maintenance of chromosomes (SMC) proteins, and are distinguished by a distinct set of three regulatory proteins named CAPG, CAPD and CAPH [5] (Figure 1A). Condensin I and II bind to different regions of chromosomes and accomplish different functions (reviewed in [2]), but how different types of condensins are specifically targeted to their chromosomal sites is currently unknown.

Condensin binding to eukaryotic genomes is tightly regulated both temporally during cell cycle and spatially along the chromosomes. Whereas purified *Xenopus* condensin I is able to bind both naked DNA and chromatin *in vitro *[6], condensin I remains cytoplasmic until the nuclear envelope breakdown, and associates with specific chromosomal regions, as observed by immunofluorescence microscopy in cultured vertebrate cells [7,8]. Condensin II, by contrast, is nuclear at all cell-cycle stages and shows a different chromosomal binding pattern compared to condensin I [7-9]. Unlike metazoans, the unicellular eukaryotes *Saccharomyces cerevisiae* and *Saccharomyces pombe* contain a single type of condensin that binds mostly intergenic regions that include RNA polymerase III transcribed genes, centromeres and ribosomal DNA (rDNA) [10,11]. Yeast condensin is recruited independently by TFIIIC to RNA polymerase III genes [10,11], and the monopolin complex to the rDNA locus [12,13]. Recruiters of metazoan condensins I and II are not well-defined.

*C. elegans* offers an excellent experimental model for studying condensin recruitment. In addition to the canonical condensins that are recruited to all chromosomes, *C. elegans* contains a third condensin (condensin I^DC^) that is targeted specifically to the X chromosome as part of the X chromosome dosage compensation complex (DCC) [9]. During embryogenesis, two zinc-finger-containing DCC subunits, SDC-2 and SDC-3, recruit condensin I^DC^ to approximately 100 recruitment sites on the X chromosome that are defined in part by a DNA sequence motif [14-18]. After recruitment, condensin I^DC^ spreads to nearby chromosomal sites in a motif-independent manner [19]. However, the recruitment mechanisms behind the binding of condensins I and II to the autosomes are not known.

To address the specificity of condensin I, II and I^DC^ targeting to the genome, we analyzed the genomic distribution of each condensin subunit in *C. elegans* mixed stage embryos using chromatin immunoprecipitation followed by high-throughput sequencing (ChIP-seq). In mixed stage embryos, more than 95% of the nuclei are in interphase, therefore we were primarily analyzing the binding and function of condensins in interphase. The binding sites of condensins coincided mostly with tRNAs, enhancers and promoters. RNA-seq analysis in *kle-2-*mutant larvae suggested that condensin II is a transcriptional repressor. We found a 'GCGC’-containing DNA sequence motif enriched at the binding sites of all condensin types. Extension of the GCGC core motif on one side by AGGG produced the X chromosome recruitment motif for condensin I^DC^. Although their chromosomal distribution is different, high-resolution binding patterns of all condensin I^DC^ and condensin II were similar, suggesting a shared binding mechanism. Indeed, we found that SDC-2 was required for binding of both condensin I^DC^ and condensin II at recruitment sites on the X. In addition, SDC-2 also recruited the cohesin loader SCC-2 (homolog of cohesin loader Scc2 in yeast, *NIPBL* in humans) to the condensin I^DC^ recruitment sites on the X, suggesting interplay between condensin and cohesin in regulating X chromosome structure for dosage compensation. We hypothesize a model in which the specificity of metazoan condensin recruitment is achieved by transcription factors (TF) that recognize specific DNA sequence motifs to recruit condensins to their chromosomal binding sites.

## Results

Condensins I and II share the two SMC subunits MIX-1 and SMC-4, and are distinguished by three non-SMC subunits (Figure 1A). Condensin I^DC^ differs from condensin I by only one subunit, the SMC-4 variant DPY-27. We used one or two different antibodies against each condensin subunit for ChIP-seq and identified those binding sites common in multiple antibodies and biological replicates (Additional file 1: Table S1). Antibody validation and expected co-immunoprecipitation interactions from the condensin holocomplex are presented in Additional file 2. Pairwise correlation of median ChIP-enrichment scores clustered condensin I-I^DC^ and II subunits separately, confirming the distribution of individual subunits between the three condensin types (Figure 1B).

### High-resolution binding patterns of the three condensin complexes are similar

*C. elegans* condensin I and II have partially overlapping but different chromosomal localizations in mitosis and meiosis [9,20-22], and condensin I^DC^ is specifically targeted to the X [23]. Therefore, we expected to find different ChIP-seq patterns for condensin I, I^DC^ and II. Instead, binding patterns of condensin I, I^DC^ and II subunits were generally similar (Figure 1C). This similarity was not due to antibody cross-reactivity, because the HCP-6 antibody, which does not show any cross-reactivity and does not immunoprecipitate any condensin I-I^DC^ subunit [9], showed extensive co-localization with condensin I-I^DC^ subunits (see HCP-6 in Figure 1C). In addition, if the overlap of condensin II was due to cross-reactivity with condensin I^DC^, we would have expected an enrichment of condensin II binding sites on the X, which was not the case (Figure 1D). Compared to condensin II, condensin I^DC^ subunits consistently had higher ChIP scores on the X, suggesting a difference in chromosomal association of the two condensin complexes as captured by ChIP (note different scales on X and chromosome I in Figure 1C). Since we performed ChIP-seq in mixed stage embryos, most of the cells (more than 95% estimated by 4',6-diamidino-2-phenylindole (DAPI) staining) were in interphase. The lack of condensin I subunits on autosomes (Figure 1C and Additional file 3: Figure S1A) and the previous observation that condensin I localizes to mitotic chromosomes after nuclear envelope breakdown [9,24] suggest that most of the ChIP-seq signal is from interphase. In support of this, condensin II was shown to be nuclear during interphase [9,24], and showed a more equal distribution of binding sites among all chromosomes (Figure 1D).

KLE-2, HCP-6 and CAPG-2 are condensin II specific, thus represent condensin II binding. Condensin I and I^DC^ share all three non-SMC subunits, so hereafter we refer to the ChIP-seq signal from DPY-26, DPY-28 and CAPG-1 as from condensin I-I^DC^. To focus on the sites that are bound as a complex, we averaged the ChIP signal from the condensin-type specific CAP subunits. In addition to using averaged data, we verified that each analysis held true with single subunits, and we present HCP-6 and DPY-28 in supplemental figures. We identified a set of high confidence condensin I-I^DC^ and II binding sites by selecting only those ChIP-seq peaks that were consistent across two or more non-SMC subunits (Figure 1D). As noted previously, 97% of condensin I-I^DC^ binding occured on the X chromosome [16,19]. Condensin II was similarly distributed between autosomes and the X. Condensin II bound to stronger condensin I-I^DC^ sites on the X chromosome (Figure 1E and Additional file 3: Figure S1B).

### Condensin binding sites are enriched at active promoters

**Figure 2:**
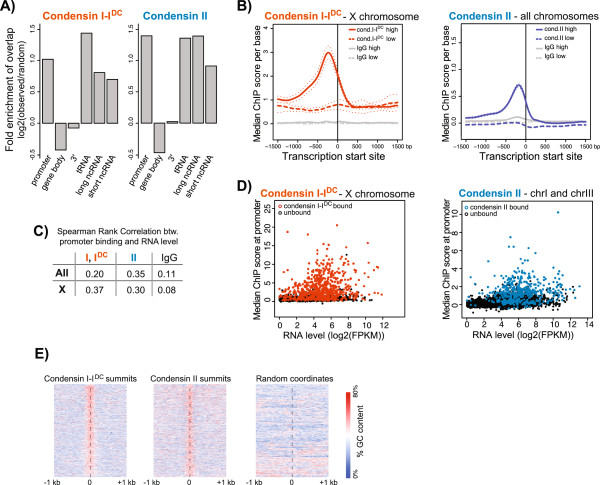
**Condensin sites are enriched at promoters and tRNAs, and binding positively correlates with transcription. (A)** Enrichment or depletion of condensin binding sites at various genomic annotations are given. Random enrichment and p-values were calculated by a permutation test randomly distributing the condensin peaks 10,000 times. For both condensin I-I^DC^ and II, there is a significant enrichment of binding sites at 1 kb promoters and near non-coding RNAs (*P* = 0.0002), depletion within gene bodies (transcription start site (TSS) to transcription end site (TES)) (*P* = 0.0002), and no significant enrichment or depletion at the 3′ of genes (*P* > 0.05). **(B)** Condensin ChIP signal is aligned at the TSSs of expressed genes (top 25% by RNA level, solid lines) and not expressed genes (bottom 25% by RNA level, dashed lines). The surrounding dots represent the 95% confidence level. As a control, immunoglobulin G (IgG) ChIP signal at the TSSs is plotted in grey. **(C)** Spearman rank correlation coefficient between median ChIP score at 500 bp promoters and RNA level at respective genes are given for the whole genome and the X chromosome. There is a slight positive correlation between condensin binding and transcription. **(D)** Median ChIP score within 500 bp upstream of the TSS is plotted against the RNA level of each gene. Condensin promoter-bound genes (defined by overlap with a condensin site within 1 kb of the TSS) are highlighted in orange (condensin I-I^DC^) and blue (condensin II). **(E)** GC content of 50 bp windows is plotted across condensin I-I^DC^ and II binding summits. As a control, the GC content over 1500 random coordinates was determined and plotted in the same way as the actual condensin summits. Average GC content is high around the peak of condensin binding.

To identify the regions where condensins preferentially bind, we analyzed condensin binding sites with respect to several genomic annotations. Condensin binding sites were significantly enriched at promoters, near tRNA genes, and non-coding RNAs (Figure 2A, Additional file 4: Figure S2). To eliminate the possibility that condensin-bound tRNA genes only occur at promoters, we removed tRNA genes that were within 1 kb of a transcription start site (TSS) and determined that the overlap of tRNA and condensin binding sites remained significant (23% and 6% of tRNAs overlapped with condensin I-I^DC^ and II sites, respectively, *P* = 0.0002). Condensin binding at tRNA genes suggests that TFIIIC-mediated condensin recruitment may be conserved between *C. elegans* and yeast [10,11].

Binding was highest within 500 bp of the TSS (Additional file 5: Figure S3A) and 45% of condensin I-I^DC^ and 62% of condensin II peaks were located at promoters. Condensin binding at promoters positively correlated with the transcriptional activity of the downstream gene (Figure 2B,C), but not all active promoters were bound (Figure 2D). Gene Ontology term analysis of bound promoters showed a slight (1.3-fold) but significant (*P* = 3.5e^-17^) enrichment for genes with embryo development function (Additional file 6: Table S2). Approximately 20% of highly expressed genes (top quartile by RNA level) had a condensin II binding site within 1 kb, and about 70% of X chromosome genes had a condensin I-I^DC^ binding site within 1 kb. Therefore, although transcriptional activity is important, it is not sufficient to explain the specificity of condensin binding at certain promoters.

We noticed that all condensin binding sites showed a prominent enrichment of GC content compared to surrounding regions and random coordinates (Figure 2E). In *C. elegans*, GC content of X chromosome promoters is higher than that of autosomal promoters [25]. It is possible that higher GC content is a DNA sequence feature for condensin binding, and X promoters evolved to contain higher GC content to support condensin I^DC^ binding.

### Condensin binding sites coincide significantly with a subset of transcription factors

**Figure 3:**
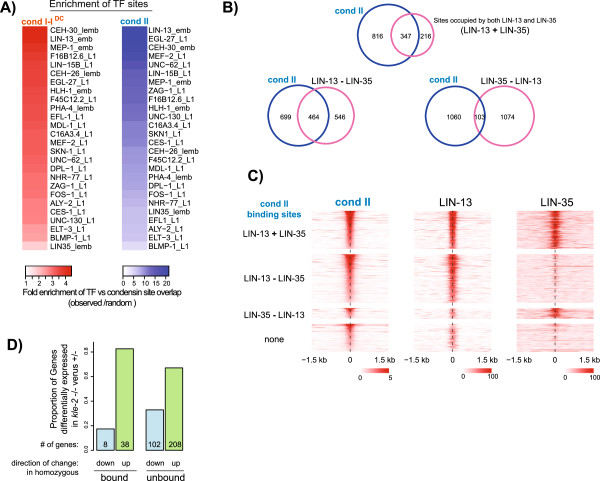
**Condensin II binding overlaps with transcription factors non-randomly, and expression analysis suggests a repressive function for condensin II. (A)** Transcription factor sites from modENCODE are ranked by the fold enrichment of overlap with condensin binding sites. Fold enrichment is determined as the ratio of percentage overlap in observed versus average of random distributions of condensin sites 10,000 times across the genome. The results indicate that condensin binding is not random with respect to TF binding sites. **(B)** Venn diagrams show the overlap of condensin II binding sites with LIN-13 (modENCODE_3342, embryos) and LIN-35 (modENCODE_3925, late embryos) (top), with LIN-13 only (bottom left), and with LIN-35 only (bottom right). The proportion of overlap between condensin II and LIN-13 and LIN-35 is higher at sites that are occupied by both proteins. The given overlap represents the number of condensin II peaks overlapping with the respective LIN-13 and LIN-35 sites. **(C)** Average ChIP-seq enrichment from condensin II, LIN-13 and LIN-35 are plotted across the summit of each condensin II peak. Condensin II binding sites are divided into four groups. The top group consists of condensin II binding sites overlapping with LIN-13 and LIN-35, the second overlaps with LIN-13 only, the third with LIN-35 only and the last group does not overlap with either LIN-13 or LIN-35. The peaks are in decreasing ordered based on their ChIP enrichment. A large number of condensin II sites show high LIN-13, but not LIN-35, binding. Condensin II binding is stronger at sites bound by both LIN-13 and LIN-35. **(D)** Differential expression analysis (RNA-seq) in *kle-2* null heterozygote versus homozygote larval stage 2/3 (L2/L3) larvae. The proportions of genes bound or unbound by KLE-2 with increase or decrease in transcript level in homozygous mutant are shown. Transcript levels of proportionally more genes increase in *kle-2* mutant. TF, transcription factor.

To determine additional factors that distinguish condensin-bound promoters, we compared condensin and TF binding sites, and found a subset of TFs that bound to the same promoters as condensins (Figure 3A). Previous studies showed that multiple TFs bind to a set of high occupancy binding sites (HOT) [26]. There was an overlap of 5% and 22% of condensin I-I^DC^ and condensin II sites with HOT sites, respectively (*P* = 0.0002). The significance of overlap with TFs remained the same when HOT sites were eliminated from the analysis (Additional file 5: Figure S3B). Percentages of overlap for each TF depended on the number of binding sites, and are shown in Additional file 5: Figure S3C. Among individual TFs, we found that 69% of condensin II sites and 53% of LIN-13 sites overlapped with each other, making LIN-13 the top TF that significantly overlapped with condensin II.

*Lin-13* has a retinoblastoma protein (pRb) interaction motif and functions in vulval development with the single pRb homolog LIN-35 in *C. elegans *[27,28]. In *D. melanogaster*, condensin II subunit dCAPD3 binding to chromatin is reduced upon pRb mutation [29]. If, in *C. elegans*, LIN-13 recruits condensin II through LIN-35, then those LIN-13 sites that are also bound by LIN-35 (576) should overlap with condensin II more than those LIN-13 sites that are not bound by LIN-35 (1,033). The overlap of LIN-13 and LIN-35 co-occupied sites with condensin II sites was only slightly higher than that of LIN-13 sites without LIN-35, 60% and 50%, respectively (Figure 3B). Conversely, overlap of LIN-35 with condensin II was higher at those sites that were also bound by LIN-13 (45%) compared to unbound sites (9%). This suggests that the potential interaction between LIN-13 and condensin II is mostly LIN-35 independent. LIN-35 functions within a conserved multi-protein complex called DRM in *C. elegans* (hDREAM in humans) that also contains EFL-1 and DPL-1 [27,28]. The 555 DRM binding sites that were bound by LIN-13 showed a greater overlap with condensin II (63%) compared to 787 DRM sites that were not bound by LIN-13 (13%). We divided the condensin II sites into four groups according to binding site overlap with LIN-13 and LIN-35 (Figure 3C). This grouping indicated that a large number of condensin II sites show high LIN-13 binding, but not LIN-35, suggesting that if pRb-mediated condensin II recruitment is conserved in *C. elegans*, LIN-35 may depend on the presence of LIN-13 for condensin II recruitment.

### Condensin II mutation causes transcriptional defects that suggest a repressive function

In yeast and *D. melanogaster*, condensin has been implicated in transcriptional repression [30-33]. One recent study in *D. melanogaster* indicated that the condensin II subunit CAPD3 is required for transcriptional activation of a cluster of antimicrobial peptide genes [34]. To understand the role of condensin II in transcription regulation, we performed RNA-seq in *kle-2* null mutant L2/L3 larvae. Maternally loaded KLE-2 allows *kle-2* null mutant (*ok1151* allele) to grow up to be sterile adults. We compared gene expression in heterozygous and homozygous *kle-2* mutants in L2/L3 larvae before the germline is proliferated, thus containing almost entirely interphase nuclei. Differential expression analysis using DESeq2 [35] identified 356 genes whose expression was significantly different in homozygote compared to heterozygote larvae (false-discovery rate < 5%; DESeq2 results are presented in Additional file 7: Table S3). The majority of differentially expressed genes increased (70%) rather than decreased (30%) in expression. Gene Ontology term analysis did not reveal a particular group of genes that were affected by the KLE-2 mutation. Similar to published data for condensin I^DC^[17], there was no direct correlation between KLE-2 binding and changes in gene expression. Importantly, among the 46 differentially expressed and KLE-2-bound genes, 83% increased in expression compared with 67% of 310 genes that were not KLE-2 bound (Figure 3D), suggesting that the direct effect of condensin II binding is largely repressive.

### Histone modifications associated with open chromatin positively correlate with condensin binding

**Figure 4:**
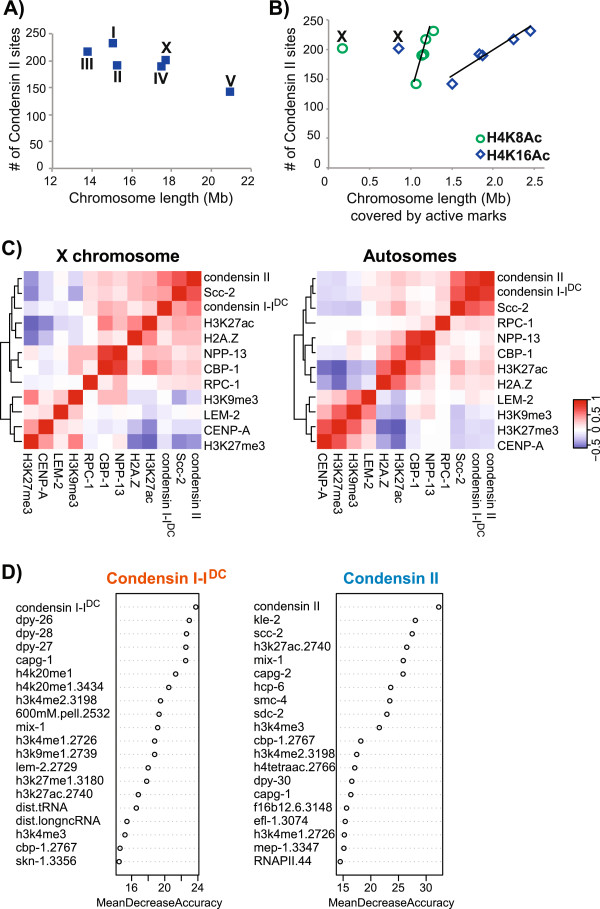
**Chromatin factors associated with open chromatin and enhancers positively correlate with condensin binding. (A)** The number of condensin II peaks are plotted with respect to the linear length of each chromosome. **(B)** The number of condensin II peaks are plotted against the length of the chromosome that is covered by H4K8Ac and H4K16Ac peaks. A trend line fitted to autosomal data indicates a positive correlation (R^2^ = 0.9). **(C)** Condensin binding within 1 kb contiguous windows across the X chromosome (left) and autosomes (right) positively correlate with active marks (for example, H3K27ac, H2A.Z) and negatively correlate with repressive marks (for example, H3K27me3, H3K9me3). **(D)** An ensemble classifier (random forests) was learned to predict condensin binding across the genome. The top 20 features (among 92 total features, Additional file 1: Table S1) with the highest predictive power are plotted for condensin I-I^DC^ (left) and condensin II (right) with the most important feature on top. The features are ranked based on the mean decrease in accuracy, which describes the difference between the error-rate of the actual classification and the error-rate after permuting the feature, averaged over all classifiers (trees).

To understand the chromatin context of condensin binding, we analyzed the relationship between condensin binding sites and chromatin features mapped by modENCODE [36]. We observed a general positive correlation between condensin binding and marks of active chromatin. In yeast, the number of condensin binding sites throughout the cell cycle directly correlates with chromosome length. The number of *C. elegans* condensin II binding sites was not proportional to chromosome length (Figure 4A), but positively correlated with the length of chromosomes associated with active histone marks (Figure 4B). The X chromosome was an exception, perhaps due to the dosage compensation mechanisms that alter its chromatin [37,38]. Condensin I-I^DC^ and II binding across the genome correlated positively with open chromatin marks such as H3K27ac and negatively with heterochromatin marks such as H3K27me3 and H3K9me3 (Figure 4C and Additional file 8: Figure S4). This correlation was at the domain-wide level (as analyzed in 1 kb windows), as we did not see a particular histone modification that peaked at condensin sites in a 'metagene’ type of analysis (data not shown). In immunofluorescence studies, centromere proteins overlapped with condensin II binding in mitotic cells [22,39]. We did not observe a positive correlation between CENP-A and condensin II ChIP-seq signals in mixed stage embryos, suggesting that, in interphase nuclei (most nuclei in mixed stage embryo cells), there is no overlap. Alternatively, given that CENP-A binds to only a small subset of the CENP-A positive regions in the genome per cell [40], the overlap of CENP-A with condensin II might only be apparent in single cells. To understand which chromatin factors best predict condensin binding, we used a machine-learning approach. In *C. elegans*, H4K20me1 is highly enriched across the X chromosome by DCC [37,38], and thus H4K20me1 was the most discriminative factor for condensin I^DC^ binding (Figure 4D). For condensin II, highly predictive chromatin features are H3K27ac and CBP, both markers of active enhancers, suggesting that condensin binding is enriched at active enhancers [41,42]. Among 201 condensin II binding sites that were 2 kb away from an annotated TSS, 58 overlapped with a CBP binding site (*P* = 0.0002).

### DNA sequence motifs enriched at condensin binding sites show specific features

**Figure 5:**
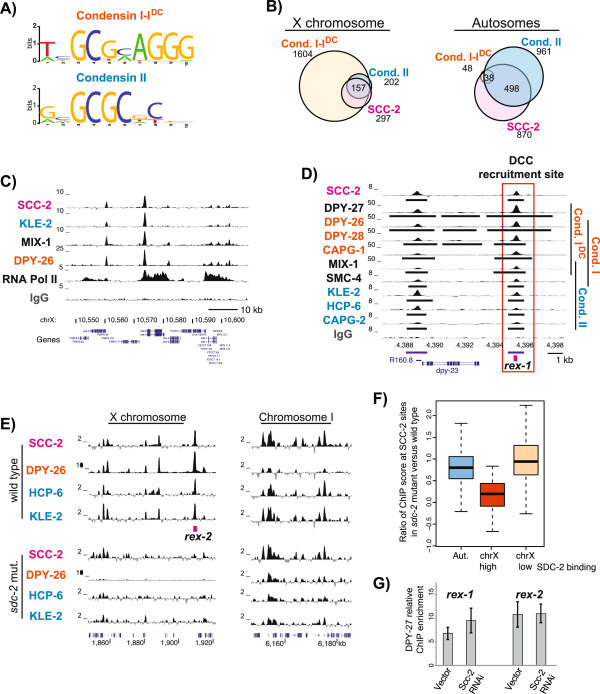
**Chromosomal recruitment of condensin I**^**DC **^**and condensin II involves both shared and distinct regulators. (A)** Motif logos of 10 bp DNA sequence motifs enriched at the top condensin sites are shown. **(B)** Overlap between binding sites of condensin I-I^DC^, condensin II and SCC-2 is shown. Numbers under each factor indicate the total number of binding sites. Overlapping numbers are based on the number of SCC-2 peaks. **(C)** University of California Santa Cruz (UCSC) browser view exemplifying SCC-2 ChIP-seq signal, which is mostly restricted to promoters. **(D)** SCC-2 and condensin II binding at a well-defined condensin I^DC^ recruitment site on the X (*rex-1*). **(E)** In *sdc-2* null mutant (TY1072), condensin I-I^DC^ (DPY-26), condensin II (HCP-6, KLE-2) and SCC-2 binding is diminished at *rex-2* (left panel), but remains largely similar on autosomes (right panel). **(F)** Box plot of the ratio of ChIP enrichment in *sdc-2* mutant versus wild type within the binding peaks of SCC-2. Binding sites are classified as being on autosomes, on the X with low SDC-2 binding and high SDC-2 binding. **(G)** qPCR analysis of DPY-27 ChIP enrichment in embryos isolated from adults that were fed vector (control) and PQN-85 RNAi. ChIP enrichment is expressed as relative to the negative control locus. Error bars are the standard deviations from three to five biological replicates. ChIP, chromatin immunoprecipitation; DCC, dosage compensation complex.

Although condensin II bound to the condensin I-I^DC^ sites on the X, condensin I-I^DC^ did not bind to most of the autosomal condensin II binding sites (Figure 1D). To understand the specificity of condensin I^DC^ and II recruitment, we searched for DNA sequence features that distinguish condensin II and condensin I^DC^ binding. Previous studies have shown that condensin I^DC^ is first recruited to approximately 100 sites, and then spreads to other chromosomal sites [19,43,44]. A 10 bp DNA sequence motif was enriched at the condensin I^DC^ recruitment sites (Figure 5A) and mutation of the motif abolished condensin I^DC^ recruitment on extrachromosomal arrays [16-18], indicating that the condensin I^DC^ DNA sequence motif plays an important role in condensin I^DC^ recruitment.

We found a GCGC-containing DNA sequence motif that was enriched under condensin II binding sites (Figure 5A). It is interesting to note that both the condensin II and condensin I^DC^ motif included the GCGC core, but the condensin I^DC^ motif was extended on one side by AGGG, suggesting that X-specificity of condensin I^DC^ recruitment is achieved by cofactors that recognize AGGG. Genome-wide, 11% of the condensin II motifs were bound by condensin II. Thus, similar to other TFs (for example, [45]), only a portion of the potential DNA sequence motifs were bound by condensin II. Other factors such as chromatin accessibility and unknown co-factors may be involved in binding specificity. Indeed, if we took those motifs that were in a 2 kb window significantly enriched for an active histone mark, such as H4K16ac, the percentage of motifs that were bound increased from 11% to 29%. In addition, similar to condensin I^DC^ motif, which is more clustered at the bound sites [16], we found that 1 kb genomic windows that contained more than one condensin II motif were around 2.5-fold more likely to be bound by condensin II, compared to those with only one motif. Therefore, motif clustering and open chromatin context help specify selection of the motifs that are bound.

Not all condensin II binding sites have the motif. At those condensin II sites without the motif, other factors may be responsible for binding. Alternatively, the low proportion of condensin II sites (27%) containing a motif may be explained by potential spreading of condensin II after recruitment. Sites of spreading are not expected to contain the motif. For example, for condensin I^DC^, a high percentage of potential recruitment sites (56%) contain the motif, but not sites of spreading (8%) [17]. A systematic analysis of the recruitment capacity of the identified condensin II sites is needed to address if there is any spreading.

### SDC-2 is required for binding of condensin I-I^DC^, condensin II and the cohesin loader to X chromosomal DCC recruitment sites

In metazoans, proteins that are involved in condensin recruitment to chromosomes are not well understood. In yeast, condensin binding overlaps with that of cohesin-loading complex Scc2/4, which increases condensin association with chromosomes [10]. To test if the overlap with Scc2/4 is conserved between yeast and *C. elegans*, we performed ChIP-seq analysis of SCC-2 (also known as PQN-85) and found that a remarkable 95% of SCC-2 sites overlapped with condensin I-I^DC^ on the X, and 60% with condensin II genome-wide (Figure 5B). The overlap remained similar when HOT regions were excluded (Additional file 9: Figure S5A). Not all condensin sites overlapped with SCC-2, suggesting that, unlike yeast, condensin does not depend on SCC-2 for binding [19,43]. SCC-2 binding was higher at promoters, and positively correlated with transcription (Additional file 9: Figure S5B). A GAGA-containing DNA sequence motif was present in 58% of SCC-2 binding sites (Additional file 9: Figure S5C). Unlike *Drosophila* Nipped-B [46]*,* and similar to *S. cerevisiae* Scc2 [10], SCC-2 binding was not high within transcribed regions, but remained intergenic (Figure 5C).

Almost complete overlap (96%) of condensin II binding sites with condensin I-I^DC^ on the X chromosome supports the existence of common mechanisms for chromosomal binding of different condensins. Since condensin II and SCC-2 bound to condensin I^DC^ recruitment sites on the X (Figure 5D and Additional file 9: Figure S5D), we hypothesized that condensin I^DC^ recruiters also recruit condensin II and SCC-2. Hermaphrodite-specific recruitment of condensin I^DC^ to the X chromosome is accomplished by SDC-2, SDC-3 and DPY-30 [14,15,44]. We performed HCP-6 and KLE-2 (condensin II), DPY-26 (condensin I-I^DC^) and SCC-2 ChIP-seq in *sdc-2* null mutant embryos. In the *sdc-2* mutant, DPY-26, HCP-6, KLE-2 and SCC-2 binding were abolished at an X-specific condensin I^DC^ recruitment site (*rex-2*) (Figure 5E). It is unclear why HCP-6 and KLE-2 binding at *rex-2* was diminished instead of resembling an autosomal condensin site. SCC-2 binding on autosomes and X chromosome sites that are independent of SDC-2 remained similar in the *sdc-2* mutant compared to sites that were bound by SDC-2 (Figure 5F). Our results suggest that SDC-2, the hermaphrodite-specific TF that recruits condensin I^DC^ to the X chromosome, also recruits condensin II and the cohesin loading complex subunit SCC-2 to the same sites (Additional file 10: Figure S6). Previous genetic studies established that an *sdc-2* null mutation does not cause embryonic lethality in males, thus the function of condensin II and SCC-2 recruitment by SDC-2 is not essential for general chromosome condensation and segregation [15,47,48]. It is possible that SCC-2 and condensin II recruitment by SDC-2 has a hermaphrodite-specific gene regulatory function.

To test if SCC-2 has an effect on chromosomal association of condensin I^DC^ at the recruitment site, we performed quantitative PCR analysis of DPY-27 ChIP in control versus SCC-2 knockdown embryos. By feeding RNAi, we were able to knockdown the levels of SCC-2 by approximately 80% (Additional file 9: Figure S5E). Upon SCC-2 knockdown, we did not see a significant change in DPY-27 binding at *rex-1* or *rex-2* (Figure 5G). Consistently, immunofluorescence analysis of condensin I and II binding on meiotic chromosomes did not show a significant difference between wild type and *scc-2* mutants [49].

## Discussion

Condensins are a major structural component of eukaryotic chromosomes, and are essential for proper chromosome condensation and segregation during mitosis and meiosis. In addition, condensins also associate with chromosomes during interphase. Yeast condensin bind to chromosomes in interphase and mitosis [10], and metazoan condensin II is nuclear throughout the cell cycle [7,8]. We know little about how condensins are targeted to and bind chromosomes. Evidence from various organisms suggests that condensin recruitment is accomplished in part by DNA-bound recruiting proteins.

In yeast, TFIIIC binds to a DNA sequence motif called the B-box element and recruits condensin [10,11]. We also observed a significant overlap between *C. elegans* condensin binding and tRNA genes (Figure 2A), suggesting that TFIIIC-mediated condensin recruitment may be conserved between *C. elegans* and yeast [10,11]. In addition to TFIIIC, other condensin recruiters must exist, because tRNA genes constitute only a small fraction of the *C. elegans* condensin I-I^DC^ and II binding sites. Evidence for multiple condensin recruiters also comes from yeast, because another DNA-binding protein, Fob1, recruits condensin to rDNA [12]. The recruiters of metazoan condensins are not known. Our data indicate that condensin I^DC^ and condensin II binding is enriched at active promoters and promoter distribution is not random, such that promoters bound by certain TFs tend to also be bound by condensins. Non-random overlap of condensin II binding with certain TFs raises the possibility that TFs may specify condensin recruitment.

Condensin II binding at promoters and enhancers may be indicative of its gene regulatory function during interphase, because condensin II was shown to play a role in gene regulation in flies [1,34]. Our gene expression analysis in *kle-2* mutant larvae indicates that condensin II regulates transcription, most likely as a repressor. Condensin I^DC^ within the DCC also represses X chromosome transcription. Although a repressor, condensin I^DC^ binding at promoters positively correlates with transcription [16]. Condensin II acts similarly in that, while binding at active promoters, the function of the complex is largely repressive. It is possible that condensin binding regulates transcriptional activity through a mechanism that does not completely silence the genes. Alternatively, binding near a certain active gene may not regulate the transcriptional activity of that gene. Indeed, for both condensin I^DC^ and condensin II, there is a lack of direct correlation between bound and repressed genes. A possibility is that condensins regulate transcription by affecting long-range interactions between promoters and enhancers. Through evolution, condensin I^DC^ may have developed this mechanism for 'fine-tuning’ gene expression, resulting in approximately two-fold repression of X in XX hermaphrodites, equalizing it to that of XO males.

In this study, we identified a DNA sequence motif enriched at condensin II binding sites that is similar to the motif defined for condensin I^DC^ (Figure 5A). While both motifs contained a GCGC core, the condensin I^DC^ motif showed additional base specificity. Although we observed a high overlap of condensin II binding sites at strong condensin I^DC^ binding sites on the X, condensin I^DC^ did not bind to most autosomal condensin II sites, highlighting the specificity of the extended condensin I^DC^ motif. Although enriched on the X, condensin I^DC^ motif was also present on autosomes. However, in the autosomal context, the motif is not sufficient to recruit condensin I^DC ^[16,17,19], suggesting that additional factors are involved in restricting condensin I^DC^ binding to the X chromosome. Similar to the condensin I^DC^ motif, not all condensin II motifs were bound by condensin II. Clustering the motif and open chromatin context improved the specification of condensin II binding.

In *C. elegans*, two zinc-finger-containing proteins, SDC-2 and SDC-3, are required for recruitment of condensin I^DC^ to the X [14,15]. It is not known if SDC-2 and/or SDC-3 directly recognize and bind to the condensin I^DC^ motif. After initial recruitment, condensin I^DC^ spreads to other sites on the X chromosomes [17,19,43]. Similarly, ectopic recruitment of yeast condensin caused spreading at a nearby site [10], suggesting that recruitment and spreading is a conserved feature of condensin binding to chromosomes. The mechanism of condensin spreading is still unknown. Fragmented evidence from yeast and humans suggests that condensins I and II bind to different histone modifications and variants [13,50], thus affinity to specific features of the underlying chromatin may be involved in condensin spreading.

## Conclusions

Our study, to our knowledge, is the first reported genome-wide binding analysis of metazoan condensin II. In addition to conserved features of binding sites such as tRNA genes, we report condensin II binding at a subset of active promoters and enhancers. Our work identified a putative DNA sequence motif and TFs that may be involved in condensin II targeting. Many important and evolutionarily conserved structural protein complexes, including condensin, cohesin and SMC5/6, regulate essential cellular processes, yet it is unknown how they are targeted to their binding sites. Our work presents a step towards understanding chromosomal targeting of chromosomal structure proteins, proposing interaction between sequence-specific motifs and TFs for the recruitment of condensin to chromatin.

## Materials and methods

### Worm strains and growth

Mixed stage embryos (wild type N2) were isolated from gravid adults by bleaching and treated with 2% formaldehyde for 30 minutes. TY1072 (*her-1(e1520) V; sdc-2(y74) X*)) is the *sdc-2* null genetically male tetraploid (AAAA XX) strain, and was grown as the wild type strain. N2 L3 worms were obtained by growing synchronous culture in liquid media. *kle*-2-mutant larvae were from the VC768 strain (*kle-2(ok1151) III/hT2[bli-4(e937) let-?(q782) qIs48] (I;III)*), which was created by the International *C. elegans* Gene Knockout Consortium. Heterozygous adults were bleached to obtain embryos, hatched in M9 and synchronized as larval stage 1s (L1s). L1s were grown at room temperature for about 20 hours. Approximately 500 GFP- (homozygous mutants) and GFP + (heterozygous) were hand picked. The larvae were washed in M9 and transferred to Trizol for RNA purification. To collect enough embryos for ChIP upon SCC-2 knockdown, 600 ml of RNAi bacteria were grown in lysogeny broth (LB) with ampicillin to optical density (OD) approximately 0.8, induced with 0.1 mM isopropyl β-D-1-thiogalactopyranoside (IPTG) for 3 hours and concentrated 130-fold to seed 6 × 10 cm plates. Synchronized N2 L1s were grown at 20°C on SCC-2 or vector containing bacteria for four days and embryos were isolated by bleaching gravid adults.

### RNA-seq and data processing

Larva and embryos in 10 volumes of Trizol (Invitrogen, Carlsbad, CA) were freeze-cracked five times, and Trizol purification was done according to the manufacturer protocol. RNA was further cleaned up using Qiagen (Venlo, The Netherlands) RNeasy kit. mRNA was purified from 5 to 10 μg of total RNA using Sera-Mag Oligo(dT) beads (Thermo Fisher Scientific, Waltham, MA). cDNA preparation was done in the presence of 2'-deoxyuridine 5'-triphosphate (dUTP) to prepare stranded RNA-seq libraries [51]. To process the raw RNA-seq data, single-end reads were aligned to the *C. elegans* genome version WS220 using tophat version v1.4.1 [52] for strand-specific reads using default parameters. Gene expression was estimated using Cufflinks version 2.0.2 [53,54] for strand-specific reads using default parameters and supplying gene annotations. The combined expression value (fragments per kilobase of transcript per million mapped reads (FPKM)) of five replicates was determined by the median of the replicates. Differential expression analysis between heterozygous and homozygous KLE-2 mutant worms was performed using DESeq2 version 1.0.12 in R version 3.0.0 [35].

### Antibodies, quantitative ChIP and ChIP-seq

Rabbit polyclonal antibodies were raised against epitopes as indicated in Additional file 1: Table S1. Antibodies with an SDI number (SDQ) were made by the modENCODE project [26]. PubMed IDs for the publications that previously characterized antibodies and validation experiments for new antibodies that were not previously reported are detailed in Additional file 2. Embryos were washed and dounce homogenized in FA buffer (50 mM HEPES/KOH pH 7.5, 1 mM EDTA, 1% Triton X-100, 0.1% sodium deoxycholate; 150 mM NaCl). 0.1% sodium lauroyl sarcosinate (sarkosyl) was added before sonicating to obtain chromatin fragments with a majority length between 200 and 800 bp. 1 to 2 mg of embryo extract and 3 to 5 ug of antibody was used per ChIP as in [16]. Half of the ChIP DNA were ligated to Illumina or home-made multiplexed adapters and amplified by PCR. Library DNA between 250 and 500 bp in size was gel purified. Single-end sequencing was performed by GAIIx or HiSeq-2000 at the University of North Carolina, Chapel Hill, NC, USA, or New York University Center for Genomics and Systems Biology, New York, NY, USA high-throughput sequencing facilities. Quantitative ChIP (qChIP) was performed with 2 out of 50 μl of ChIP and Input DNA that was isolated from 5% of the ChIP. KAPA SYBR FAST Roche LightCycler 480 2X qPCR Master Mix (Kapa Biosystems, MA) was used in 20 μl reactions that were analyzed in a Roche LightCycler. The DNA sequence for the PCR primers are given in the last page of Additional file 2.

### ChIP-seq data processing

We aligned 28 to 50 bp single-end reads to the *C. elegans* genome version WS220 using bowtie version 0.12.7 [55], allowing two mismatches in the seed, returning only the best alignment, and restricting a read to map to at most four locations in the genome. Mapped reads from ChIP and input were used to call peaks and obtain read coverage per base using MACS version 1.4.1 [56] with default parameters. Coverage per base was normalized to the genome-wide median coverage (excluding the mitochondrial chromosome). Final ChIP enrichment scores per base were obtained by subtracting matching input coverage. Replicates were merged by averaging coverage at each base position. Reads from *sdc-2* mutants were processed separately for the X chromosome and autosomes (due to having half the amount of X reads compared to autosomes) and final ChIP enrichment scores were combined after normalization. For wild type and *sdc-2* mutant comparisons, the data sets were standardized by z-score transformation of the ChIP enrichment values based on the mean and standard deviation of data outside the peak regions, the presumed background. Raw data files and wiggle tracks of ChIP enrichment per base pair, and RNA-seq FPKM values per gene are provided at Gene Expression Omnibus database [57] under accession number [GEO:GSE45678]. For those datasets from modENCODE, Data Coordination Center accession numbers are given in Additional file 1: Table S1.

### ChIP peak finding

To determine a set of peaks per subunit, reads from the replicates were combined using the BEDTools utility mergeBam version 2.13.4 [58] and MACS was used to call peaks at *p-*value cutoff e^-10^. Only those peaks from the combined set that were also present in the majority of the individual replicates were included in the final peak set. To get final peak sets representing condensin I-I^DC^ and condensin II, we determined each base pair covered by the peaks of at least two of the three non-SMC subunits. Peak summits were determined as the position with the maximum ChIP enrichment score. To avoid penalization of long peaks with multiple summits, peaks were split into smaller peaks using PeakAnalyzer version 1.4 [59], with the minimum height being equal to the median coverage at all determined summits of the given data set and a separation float of 0.85.

### Correlation and histone modification analysis

Histone modification data was obtained from modENCODE (Additional file 1: Table S1). If available, combined wiggle files for all replicates were downloaded, otherwise all replicates were downloaded individually and averaged at each base pair. For each data set, the median ChIP enrichment score in 1 kb windows along the genome was determined. The Pearson correlation coefficient was then calculated among all data sets and a heatmap of all correlation coefficients was plotted in R version 2.15.2 using the package gplots. Hierarchical clustering was applied to the correlation matrix. A machine learning system was set up to be trained with various histone modifications, condensin subunits and presence of identified motifs. The genome was divided into 250 bp consecutive windows and those windows bound by condensin I-I^DC^ and II were determined. A stratified classification task was set up for two classes, being bound or not bound by condensin, and random forests was employed as an ensemble classifier. We trained 10,000 decision trees using the package randomForest [60] in R. Factors with the best discriminative behavior were determined by identifying those factors with the highest decrease in accuracy.

### Heat maps across genome coordinates

The binding profiles across condensin I-I^DC^ and condensin II summits were determined across a 1.5 kb window around each summit. Summits were ordered according to the ChIP enrichment score at the peak summit in decreasing order from top to bottom. The median ChIP enrichment score within 50 bp windows was plotted in R version 2.15.2 using the package gplots. We determined the GC content at each base pair using a sliding window of 15 bp along the genome. The mid position within each window was assigned the GC content of that window.

### Motif analyses

DNA sequence of ±100 bp around the summit of the top 200 ChIP binding peaks were used to identify potential binding motifs using MDScan [61]. The position weight matrix of the top identified motif was then used to identify genome-wide binding sites using TRAP [62,63].

### Overlaps between condensins and annotations

Transcript coordinates are based on Wormbase WS220. For genes with multiple transcripts, the outmost coordinates of all transcripts were defined as the coordinates for the gene*.* Non-coding RNAs (ncRNAs) were defined as long or short based on a 200 bp cutoff. The region ±200 bp around the condensin peak summit was used to identify overlaps with annotated genes. Promoters and 3′ regions were defined as 1 kb upstream of the TSS or 1 kb downstream of the transcription end site (TES), respectively. For non-coding RNAs, 1 kb around the TSS and TES was used. Overlaps were determined by the BEDTools utility intersectBed [58] with a minimum overlap of 1 bp. To identify the significance of the overlap, condensin binding sites were shuffled randomly 10,000 times and the actual overlap was compared to the average overlap of random shuffling. To analyze overlaps with TFs, TF binding sites were downloaded from modENCODE experiment ChIP-Seq Identification of *C. elegans* TF Binding Sites (Additional file 1: Table S1). Only TF binding sites from embryos and L1 stage were taken into consideration.

## Abbreviations

Bp: Base pair; ChIP-seq: Chromatin immunoprecipitation followed by high-throughput sequencing; DCC: Dosage compensation complex; FPKM: Fragments per kilobase of transcript per million mapped reads; GFP: Green fluorescent protein; HOT: High occupancy target sites; PCR: Polymerase chain reaction; pRb: Retinoblastoma protein; qChIP: Quantitative ChIP; rex: Recruitment element on the X; SMC: Structural maintenance of chromosomes; TES: Transcription end site; TF: Transcription factor; TSS: Transcription start site.

## Competing interests

The authors declare that they do not have any competing interests.

## Authors’ contributions

AK carried out the data analysis and helped to draft the manuscript. CJ and LHW carried out ChIP experiments. SEA and MK carried out RNA-seq experiments. SE conceived of the study, participated in its design and coordination and helped to draft the manuscript. All authors read and approved the final manuscript.

## Supplementary Material

Additional file 1: Table S1 Contains the list of all data sets with their GEO accession numbers, and modENCODE data coordination center numbers. Click here for file

Additional file 2 Contains the antibody validation experiments, and primers for ChIP real time PCR analysis. Click here for file

Additional file 3: Figure S1 **(A)** Distribution of condensin subunit ChIP-seq peaks across the whole chromosome IV and the X chromosome is shown. **(B)** Average ChIP-seq enrichment score from non-SMC subunit of condensin I-I^DC^ (DPY-28) and II (HCP-6) are plotted across the summit of each DPY-28 or HCP-6 binding peak. The peaks are ordered by ChIP enrichment (highest ChIP value on top), to illustrate that data from individual subunits agrees with averaged data shown in Figure 1E. Click here for file

Additional file 4: Figure S2 **(A)** Enrichment or depletion of DPY-28 and HCP-6 binding sites at various genomic annotations are given. Random enrichment and *p-*values were calculated by a permutation test randomly distributing the condensin peaks 10,000 times. The data from individual subunits agrees with that shown for combined condensin I-I^DC^ and condensin II sites in Figure 2A. **(B)** The overlap of condensin sites with various genomic annotations is shown separately for the X and autosomes. Note that the overlaps are not exclusive, and a peak can overlap with multiple annotations and vice versa. Genes represent all coding genes. Non-coding RNAs (ncRNAs) include all non-coding genes excluding tRNAs. Short and long classification is based on a 200 bp cutoff. Intergenic is defined as 1 kb away from any annotated gene, including coding and noncoding genes. 'Annotation coverage’ is the percentage of the genome covered with the annotation based on the criteria applied for overlap (for example, 1 kb promoter). Click here for file

Additional file 5: Figure S3 **(A)** ChIP enrichment of individual subunits is averaged across TSSs, indicating an enrichment of binding at promoters for each subunit. **(B)** Transcription factors (TFs) whose sites are reported at the modENCODE consortium data coordination center are ranked by the fold enrichment of overlap with condensin binding sites. Those TF sites and condensin sites that overlap with HOT regions are removed from this analysis. Fold enrichment is calculated by random redistribution of condensin binding sites 10,000 times across the genome and calculating the ratio of percent overlap in observed versus average of random distributions. **(C)** Percent overlap between TFs and condensins. For 'condensin’ columns, data indicate percentage of condensin sites that overlap with each TF (rows). For columns labeled 'TF’, data indicate percentage of TF sites that overlap with condensin. Data on the left panel for condensin I-I^DC^ is from the X chromosome only. Data on the right panel for condensin II is from the whole genome. Click here for file

Additional file 6: Table S2 Contains Gene Ontology term analysis of genes bound by condensin II. Click here for file

Additional file 7: Table S3 Contains differential expression analysis of *kle-2* mutant larvae. Click here for file

Additional file 8: Figure S4 Pearson correlation coefficients of ChIP enrichment at 1 kb windows across the genome. The numbers near labels are the modENCODE DCC IDs for the datasets. Click here for file

Additional file 9: Figure S5 **(A)** Overlap between binding sites of condensin I-I^DC^, condensin II and SCC-2 is shown. Numbers under each factor indicate the total number of binding sites. Overlapping numbers are based on the number of SCC-2 peaks. Those binding sites that overlap with HOT regions are removed from this analysis. **(B)** The median SCC-2 ChIP signal is plotted over the TSS and TES of all annotated genes. SCC-2 ChIP signal is enriched at promoters, and is proportional to transcription. Genes were ranked into five groups (highest expressed, 1^st^ quintile; lowest expressed, 5^th^ quintile) based on RNA level. As a control, IgG ChIP signal was also plotted across the transcription start and end sites (panels below). **(C)** Top 200 autosomal SCC-2 sites were used for motif search with MDScan [61]. The motif logo was created by web logo [64]. This motif is present in 58% of the SCC-2 peaks. **(D)** Heat maps demonstrating ChIP enrichment across binding peak summits for condensin or SCC-2. The peaks are ordered from strongest (top) to weaker binding (bottom). Condensin I-I^DC^ ChIP signal is high across all SCC-2 peak summits on the X (top left panel). By contrast, SCC-2 ChIP signal is high for the subset of strongest condensin I-I^DC^ binding sites, which include the majority of condensin I^DC^ recruitment sites (top right panel). The heat map indicates that condensin II is enriched at SCC-2 sites (bottom left panel) and SCC-2 is enriched at condensin II sites (bottom right panel). **(E)** Western blot analysis of SCC-2 amount in embryos used for quantitative ChIP (qChIP) (Figure 5G). The percent reduction in SCC-2 amount was calculated based on tubulin loading control and the control RNAi. Click here for file

Additional file 10: Figure S6 Hypothetical model for condensin binding. Condensins are recruited to chromosomes at sites specified by DNA sequence motifs (colored boxes). These motifs may be recognized by recruiters and cofactors that can interact with condensins. For example, condensin I^DC^ is able to interact with X-specific condensin recruiters, but not autosomal recruiters. There exists more than one condensin recruiter, since SDC-2 is required to bring condensin II to condensin I^DC^ sites on the X chromosome but condensin II is independently recruited to autosomes. After recruitment, condensins spread onto nearby chromatin (indicated by arrows). For condensin I^DC^, at least 88% of the approximately 1,600 binding sites on the X are predicted to arise from spreading. The potential role of why SCC-2 is also recruited to the condensin I^DC^ binding sites by SDC-2 is not clear. Click here for file
