# Supplementary material for: Genome-wide analysis of condensin binding in Caenorhabditis elegans
Source: Genome Biol. 2013 Oct 14;14(10):R112. doi: 10.1186/gb-2013-14-10-r112 (PMC3983662; doi:10.1186/gb-2013-14-10-r112)
Supplement: Additional file 2 — Contains the antibody validation experiments, and primers for ChIP real time PCR analysis. [file gb-2013-14-10-r112-S2.pdf]

Antibodies used

Target	Antibody	Pubmed PMID	Novus Biologicals Cat #
MIX-1	JL0004	19853451	
MIX-1	SDQ4107		
MIX-1	SDQ3892		
DPY-26	JL00003	19853451	
DPY-26	SDQ4496		
DPY-26	SDQ4561		
CAPG-1	SDQ3967		48460002
CAPG-1	SDQ3990		53230002
DPY-28	SDQ4564		
DPY-28	KH-DPY28	19119011	
DPY-27	JL00001	17293863	
DPY-27	SDQ3995		
SMC-4	OD0039		
SMC-4	KH-SMC4	19119011	
KLE-2	SDQ3898		
KLE-2	SDQ3942		48530002
HCP-6	KH-HCP6	19119011	
CAPG-2	SDQ4484		
CAPG-2	SDQ4567		
PQN-85	SDQ4481		53320002
AMA1	Q2358		38520002
AMA1	Q2357		
HTZ1 BK	BK00001_HTZ1	18787694	

For antibodies that are not characterized before, we used western blot, immunoprecipitation analysis and RNAi for validation. The following slides summarizes information for each antibody and shows co-immunoprecipitation between condensin subunits. If an antibody immunoprecipitated the correct target as a single or the primary band on a western blot, and the signal is specific tested by immunoprecipitation, and/or knockdown by RNAi, and the ChIP-seq data is reproducible and correlates with ChIP-seq data from an independent antibody to the same target (if available), then the data from that antibody was used.

DPY-27

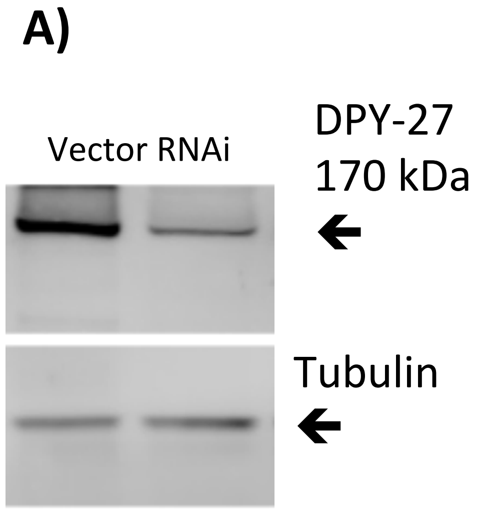

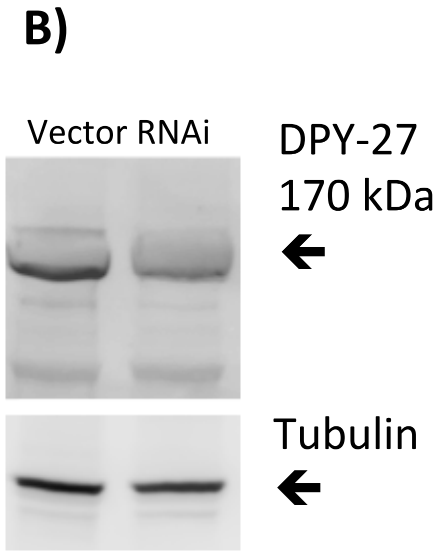

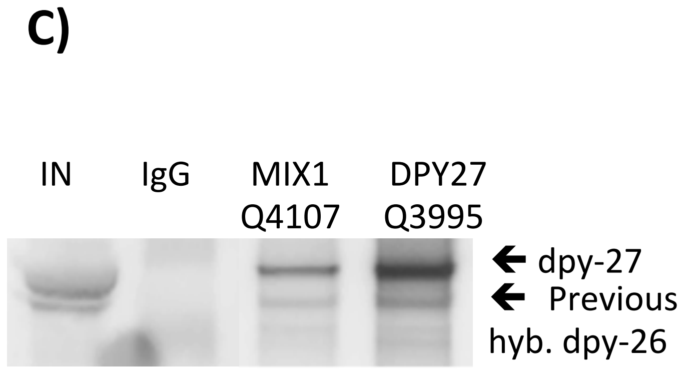

Figure 1: RNAi depletion of DPY-27 show reduction in DPY-27 signal using
A) DPY-27 JL and **B)** SDQ3995 antibodies. **C)** DPY-27 SDQ3995 antibody immunoprecipitates DPY-27 and DPY-26.

CAPG-1

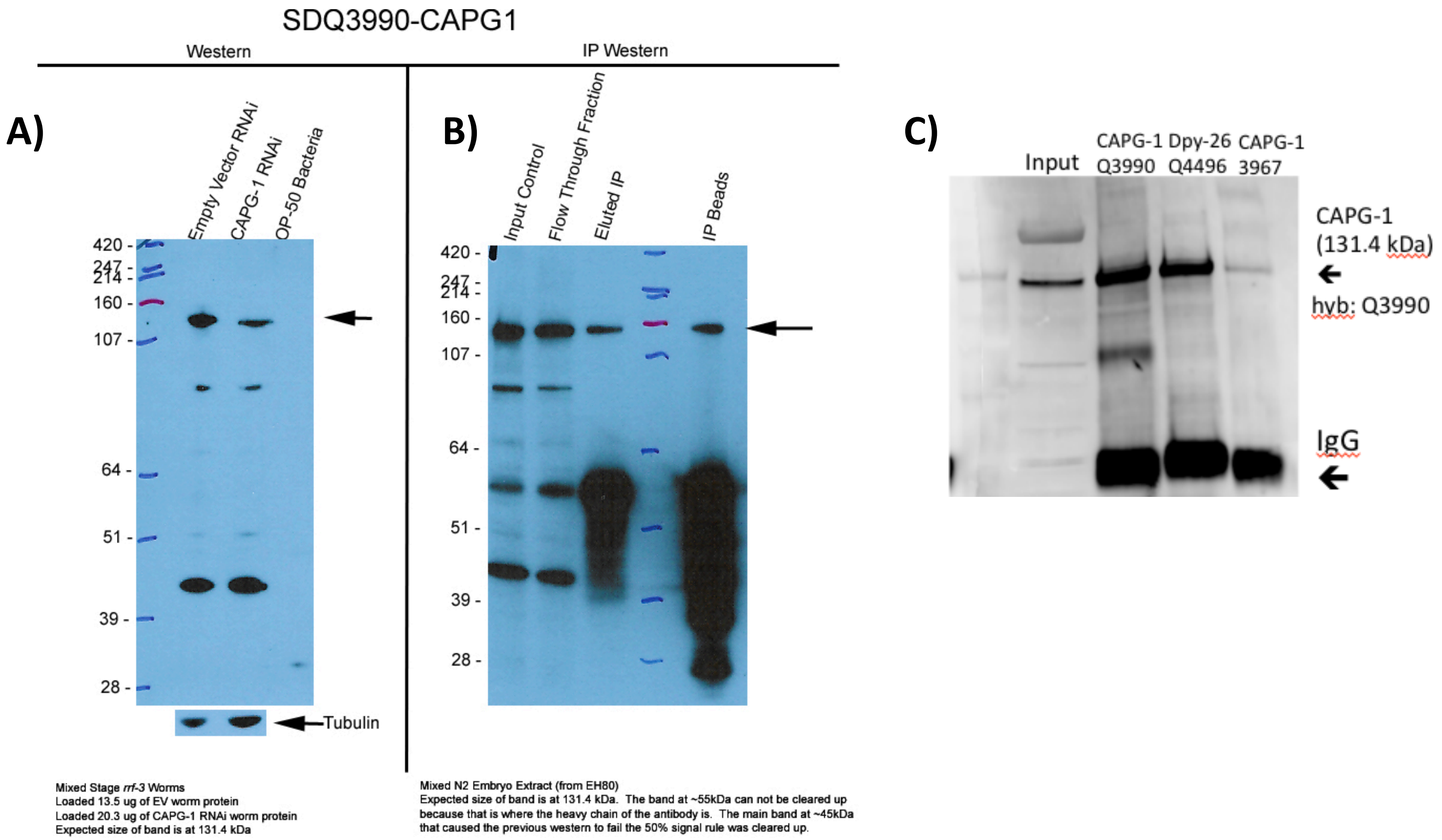

Figure 2: **A)** RNAi depletion of CAPG-1 show reduction in SDQ3990 signal. **B)** SDQ3990 and **C)** CAPG-1 SDQ3967 immunoprecipitates CAPG-1.

DPY-28 and HCP-6

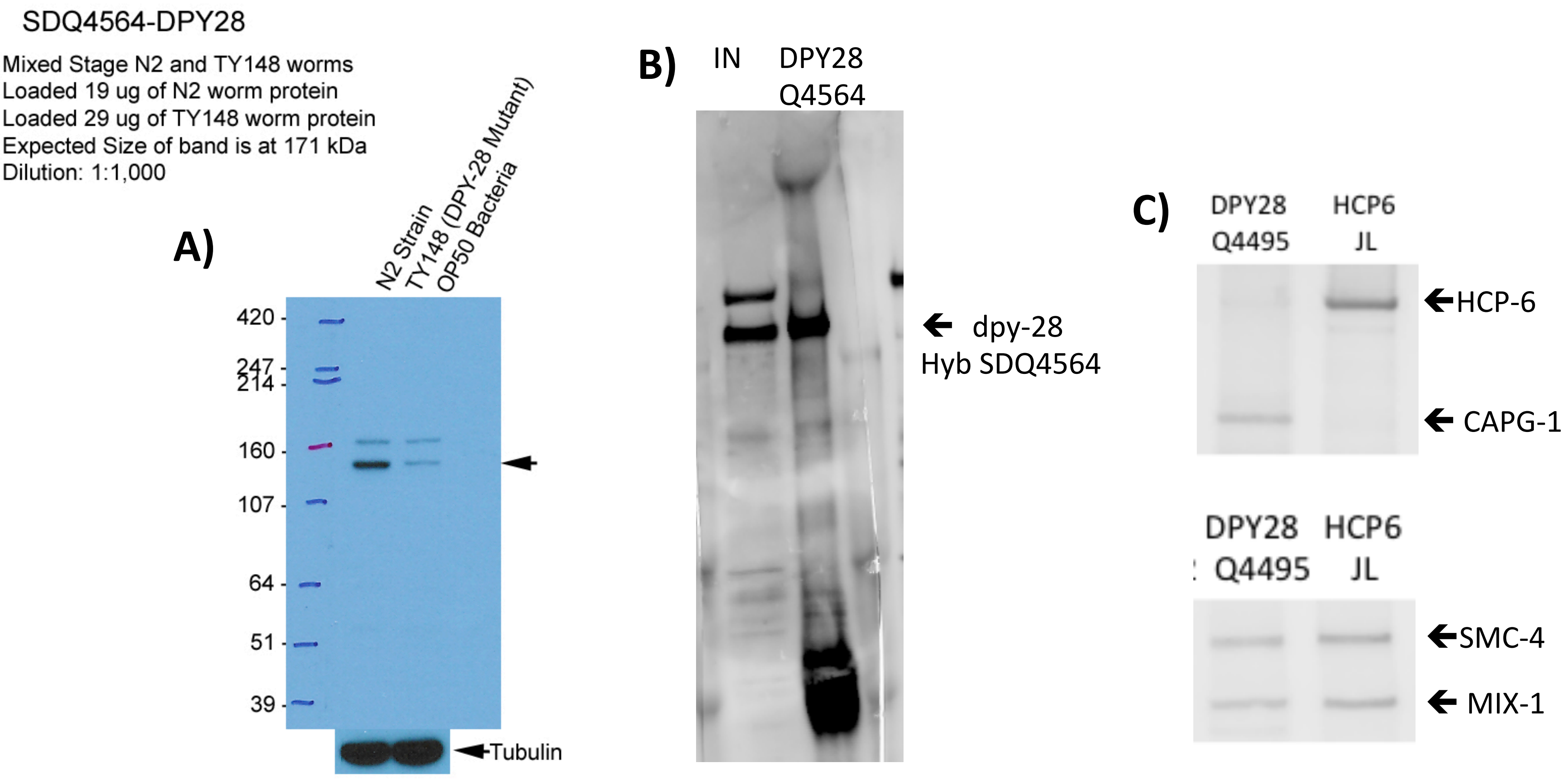

Figure 3: **A)** RNAi depletion of DPY-28 shows reduction of one band in SDQ4564 signal. **B)** DPY-28 SDQ4564 immunoprecipitates DPY-28 band and not the second band that is in the input. **C)** DPY-28 SDQ4495 does not immunoprecipitate HCP-6. HCP-6 JL immunoprecipitates SMC-4 and MIX-1, but does not immunoprecipitate condensin I-I^{DC} subunit CAPG-1.

DPY-26

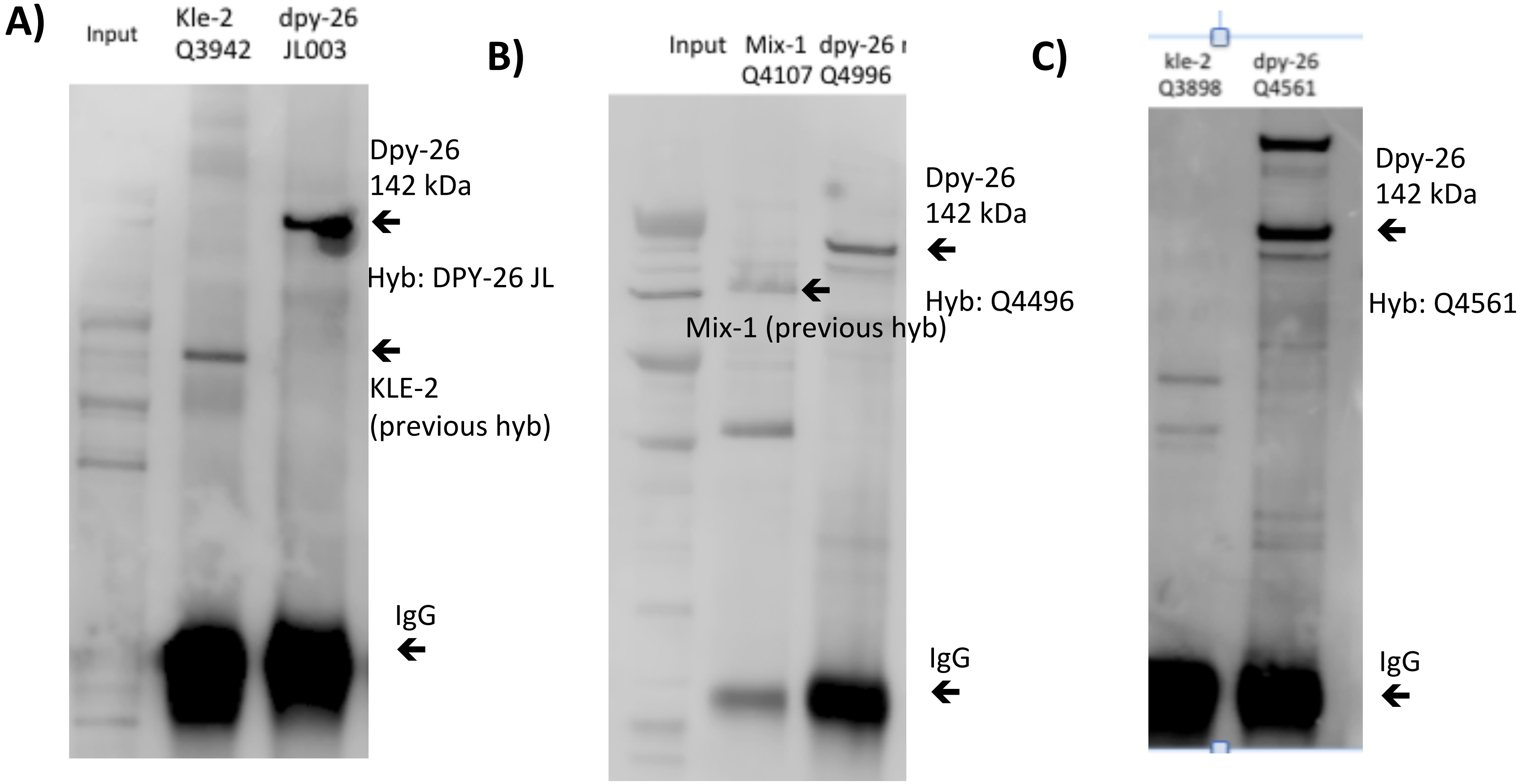

Figure 4: **A)** DPY-26 JL immunoprecipitates DPY-26. KLE-2 SDQ3942 does not immunoprecipitate condensin I-IDC subunit DPY-26. **B)** DPY-26 SDQ4496 immunoprecipitates specifically DPY-26. **C)** DPY-26 SDQ4561 immunoprecipitates DPY-26 but there is a non specific band for this particular antibody.

CAPG-2

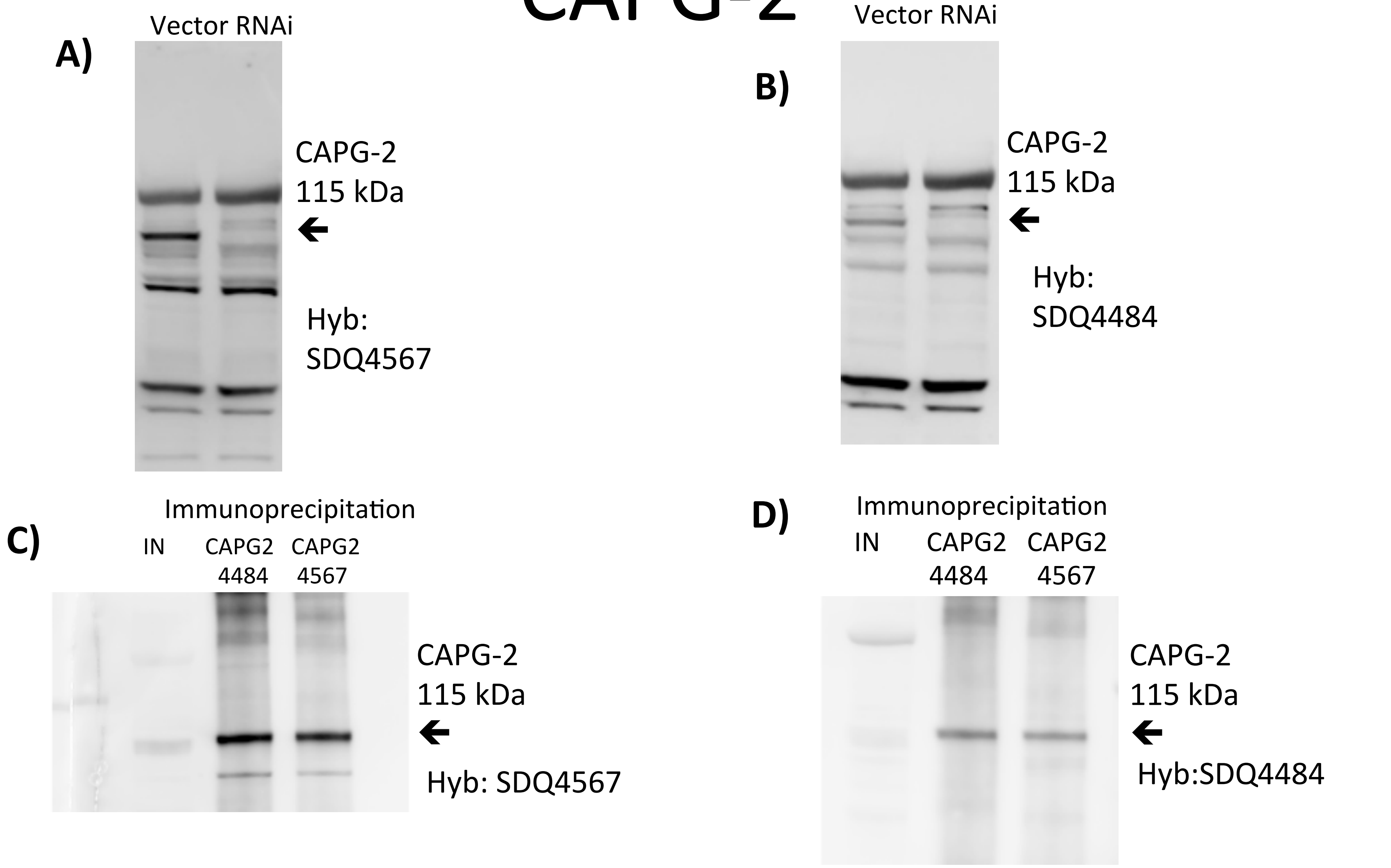

Figure 5:RNAi depletion of CAPG-2 shows reduction in **A)** SDQ4567 and **B)** SDQ4484 signal. **C)** SDQ4567 and **D)** SDQ4484 immunoprecipitate CAPG-2. CAPG-2 SDQ4567 shows a background band that is not there for SDQ4484.

KLE-2

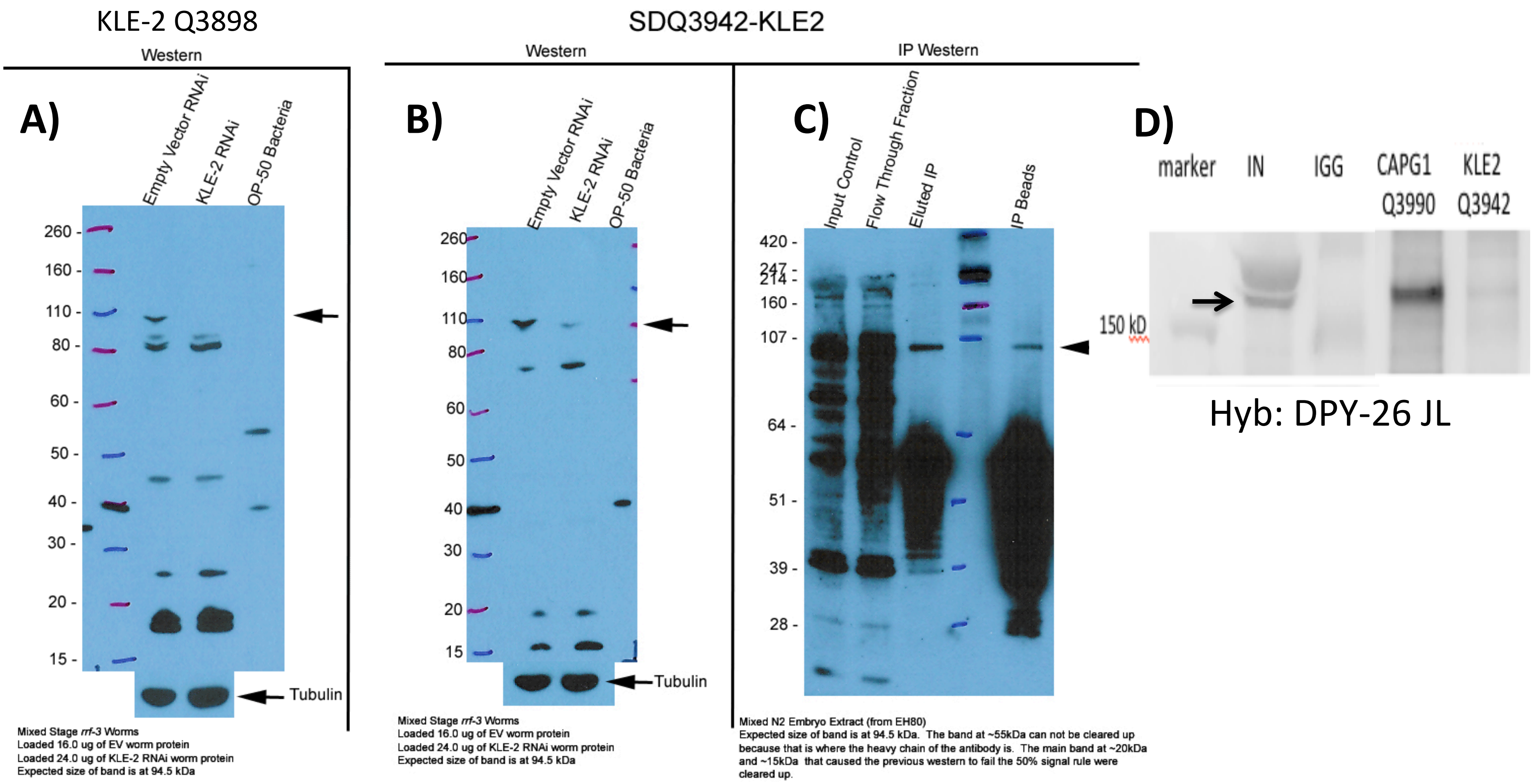

Figure 5:RNAi depletion of KLE-2 shows reduction in **A)** SDQ3898 and **B)** SDQ3942 signal. **C)** SDQ3942 immunoprecipitate specifically KLE-2 **D)** , and not DPY-26.

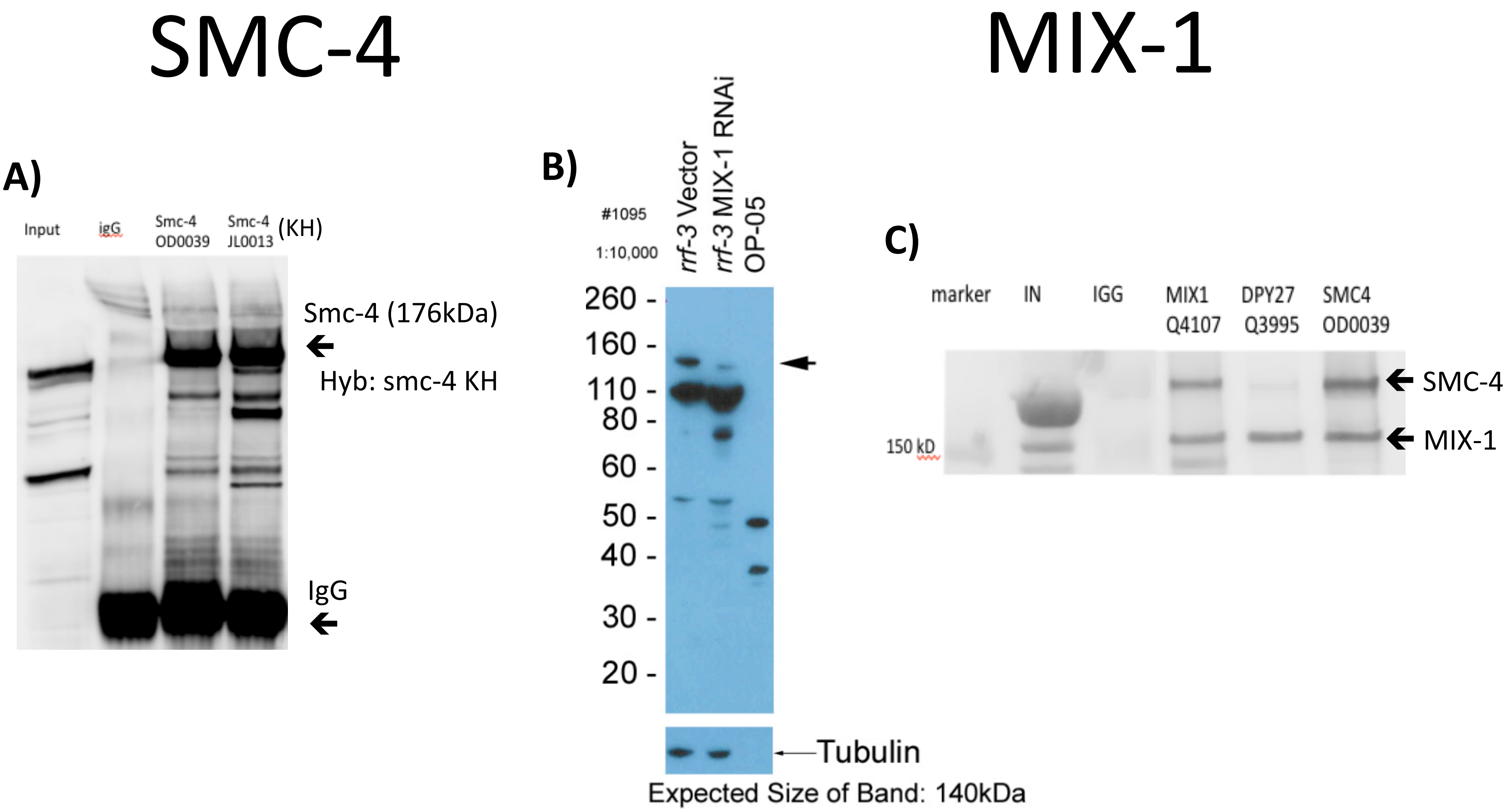

Figure 6:**A)** SMCOD0039 immunoprecipitates SMC-4. SMC-4 KH is previously characterized (see supplemental table 1). **B)** RNAi depletion of MIX-1 shows reduction in MIX-1 signal in SDQ4107. **C)** SMC-4 and MIX-1 co-immunoprecipitate.

PQN-85 (SCC-2)

SDQ4481-PQN85

Expected size of band: 252.8 kDa
1:5000 dilution
rrf-3 worms harvested at 1st generation gravid

A)

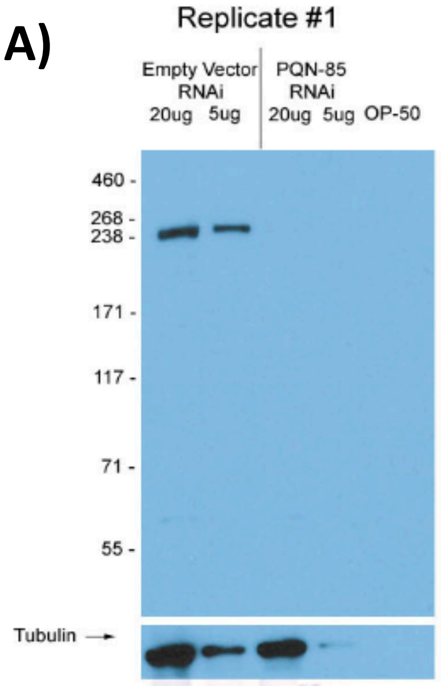

B)

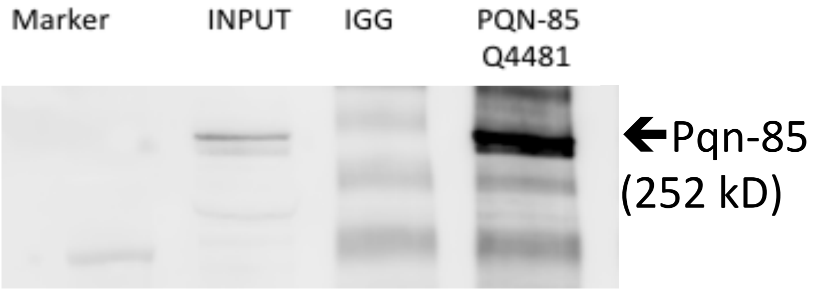

Figure 7: **A)** RNAi depletion of PQN-85 shows reduction in PQN-85 signal in SDQ4481.
B) SDQ4481 immunoprecipitates PQN-85.

qPCR primers:

GCCCGTCTATGTCGTCTCTT	GCAGTCCTCCTCATCCTTCA	dpy-23 promoter
CGTGCGTACAAAAGGAGACA	CTTTCCCCTGCCCAATTACC	rex-1
AGAAAATGCGCACACAAACG	TAATGTTTAGGCGGCAGGGA	rex-2
AGTTTGCAATCGGTGTGTCG	GTCGAGCACTTGTGAGGAAG	negative control
