# Supplementary figures and images for: Genome-wide analysis of condensin binding in Caenorhabditis elegans
Source: Genome Biol. 2013 Oct 14;14(10):R112. doi: 10.1186/gb-2013-14-10-r112 (PMC3983662; doi:10.1186/gb-2013-14-10-r112)

Supplemental Figure 1

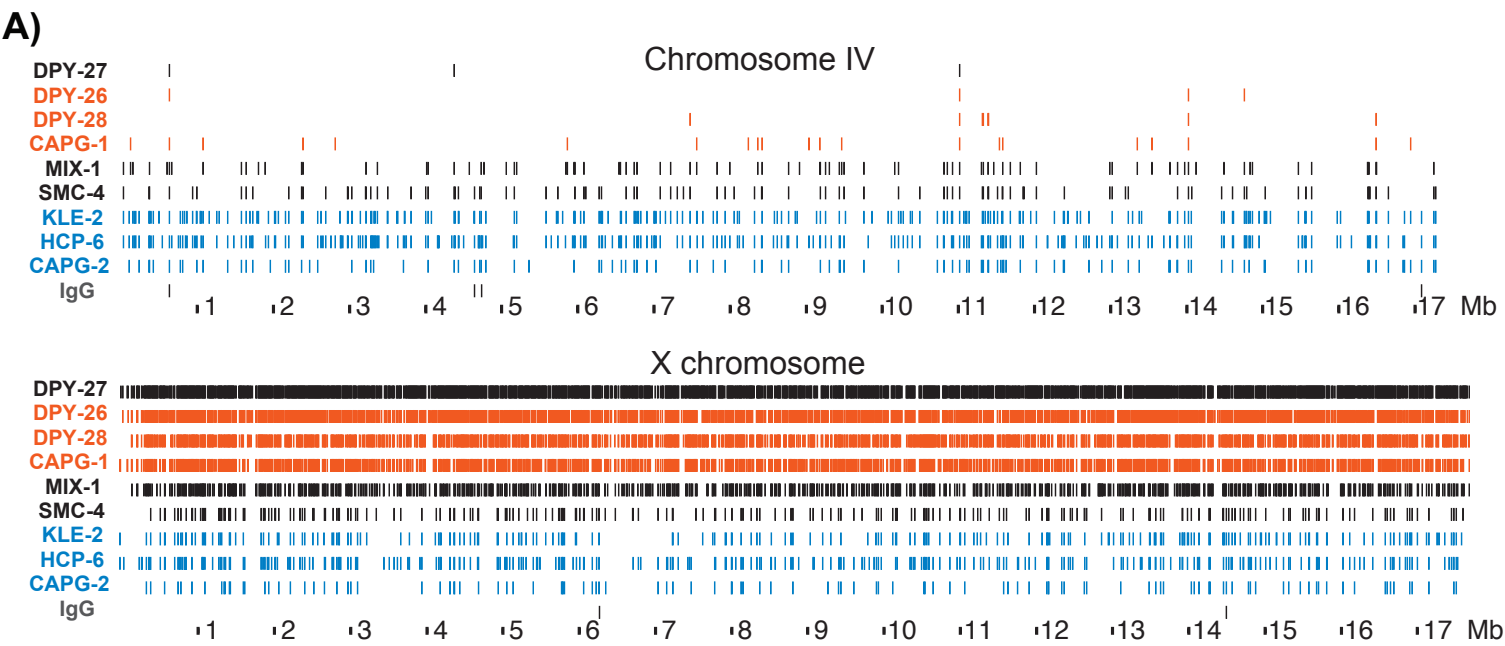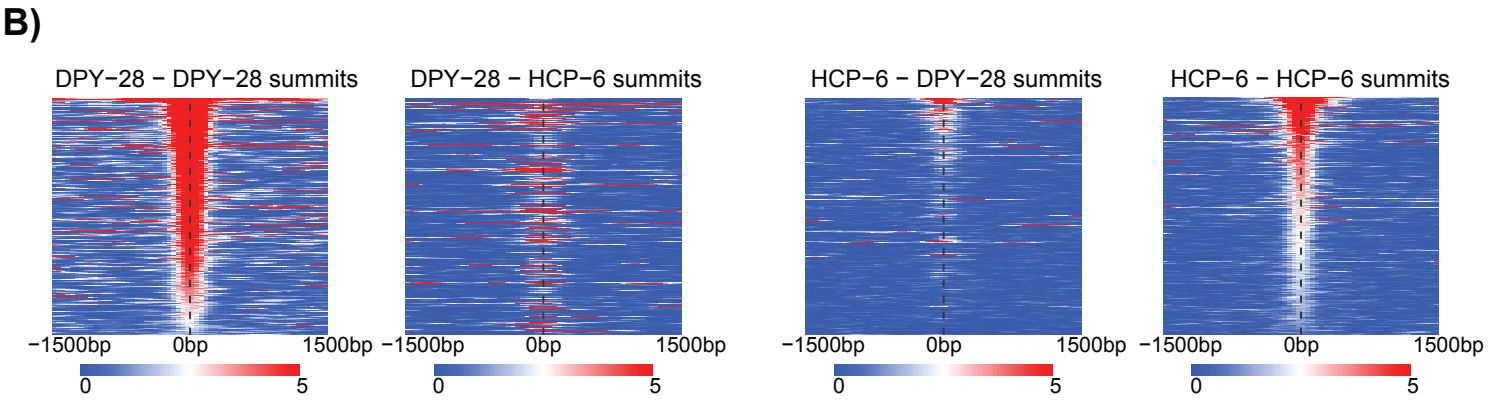

Supplement: Additional file 3: Figure S1 — (A) Distribution of condensin subunit ChIP-seq peaks across the whole chromosome IV and the X chromosome is shown. (B) Average ChIP-seq enrichment score from non-SMC subunit of condensin I-IDC (DPY-28) and II (HCP-6) are plotted across the summit of each DPY-28 or HCP-6 binding peak. The peaks are ordered by ChIP enrichment (highest ChIP value on top), to illustrate that data from individual subunits agrees with averaged data shown in Figure 1E. [file gb-2013-14-10-r112-S3.pdf]

Supplemental Figure 3

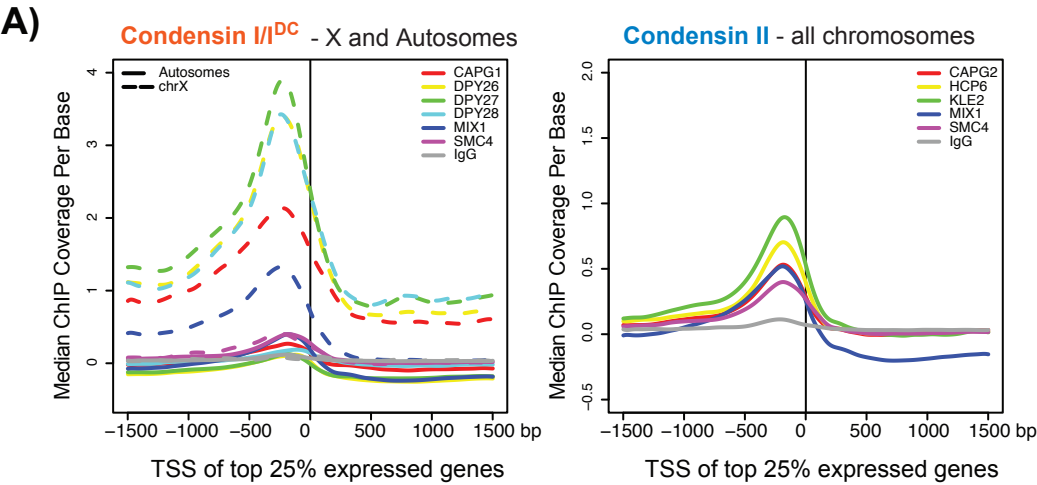

**B)**

Enrichment of TF sites - HOT sites

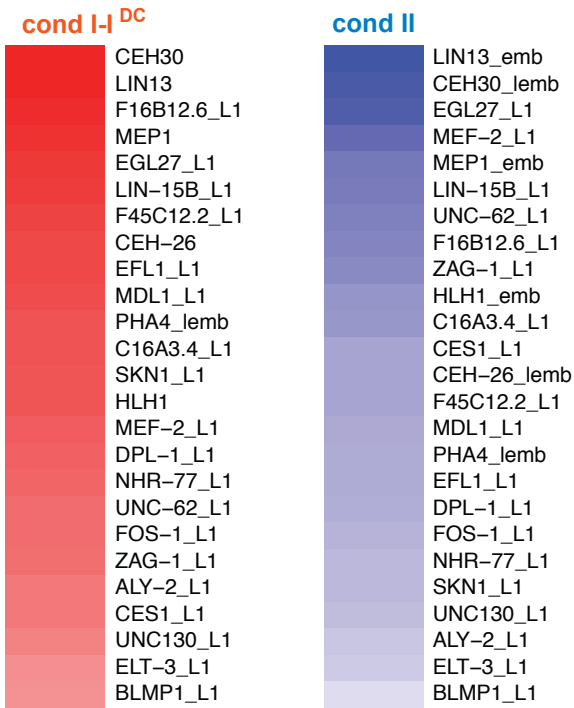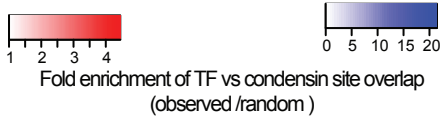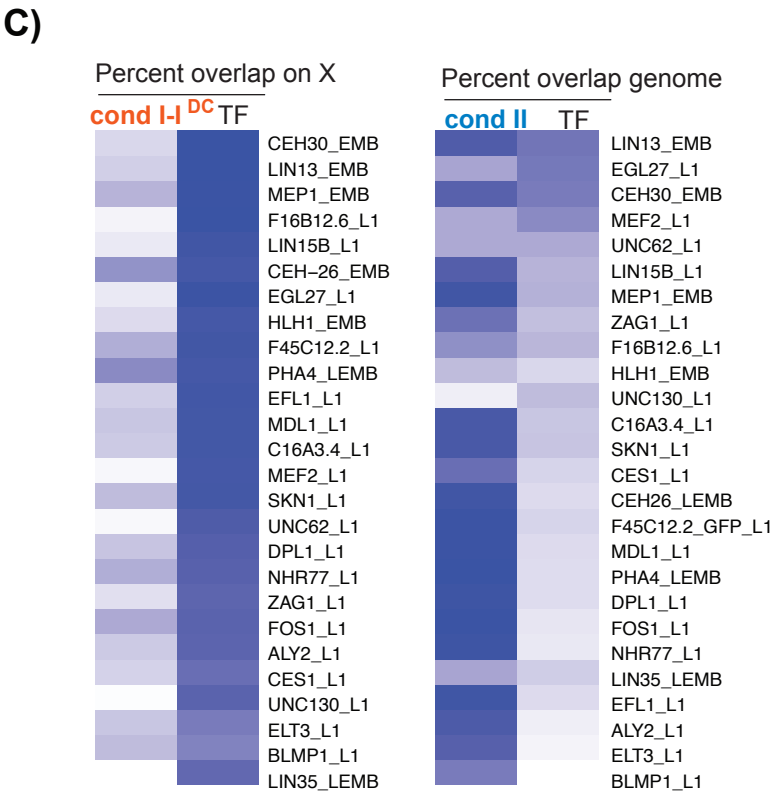

Supplement: Additional file 5: Figure S3 — (A) ChIP enrichment of individual subunits is averaged across TSSs, indicating an enrichment of binding at promoters for each subunit. (B) Transcription factors (TFs) whose sites are reported at the modENCODE consortium data coordination center are ranked by the fold enrichment of overlap with condensin binding sites. Those TF sites and condensin sites that overlap with HOT regions are removed from this analysis. Fold enrichment is calculated by random redistribution of condensin binding sites 10,000 times across the genome and calculating the ratio of percent overlap in observed versus average of random distributions. (C) Percent overlap between TFs and condensins. For 'condensin’ columns, data indicate percentage of condensin sites that overlap with each TF (rows). For columns labeled 'TF’, data indicate percentage of TF sites that overlap with condensin. Data on the left panel for condensin I-IDC is from the X chromosome only. Data on the right panel for condensin II is from the whole genome. [file gb-2013-14-10-r112-S5.pdf]

Supplemental Figure 4

ChIP Correlation within 1 kb windows across the genome

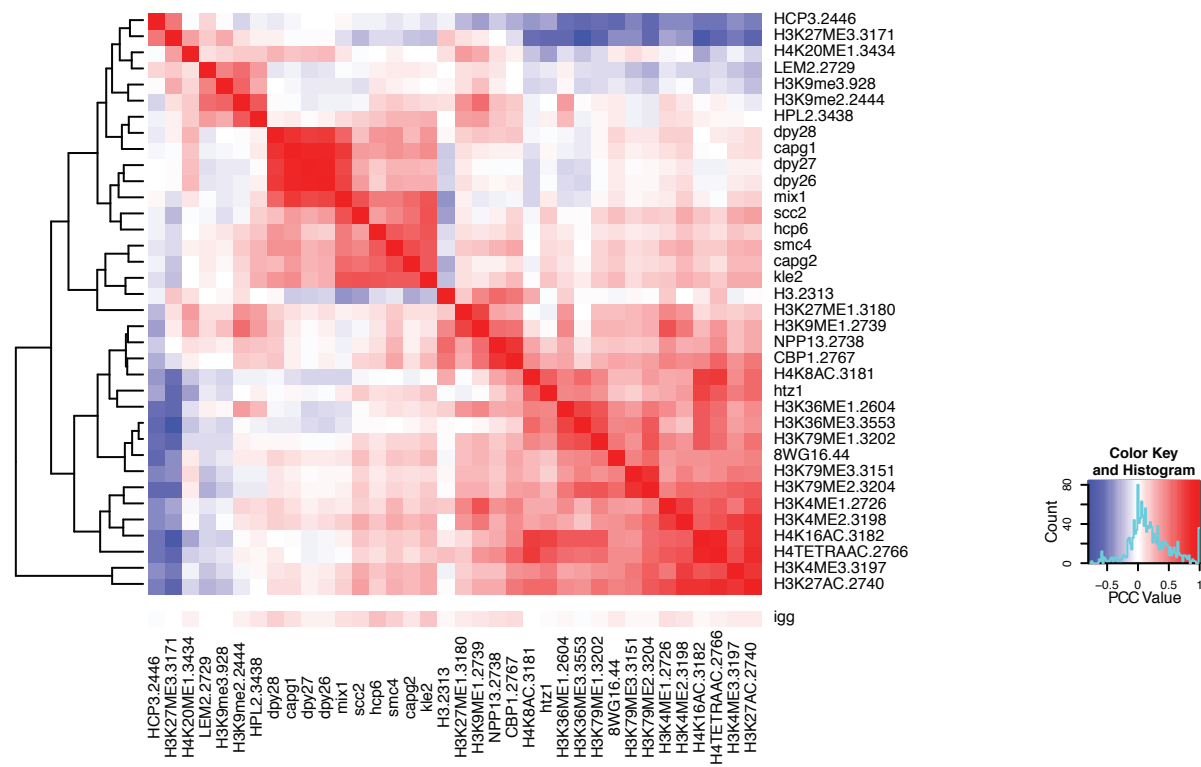

Supplement: Additional file 8: Figure S4 — Pearson correlation coefficients of ChIP enrichment at 1 kb windows across the genome. The numbers near labels are the modENCODE DCC IDs for the datasets. [file gb-2013-14-10-r112-S8.pdf]

Supplemental Figure 5

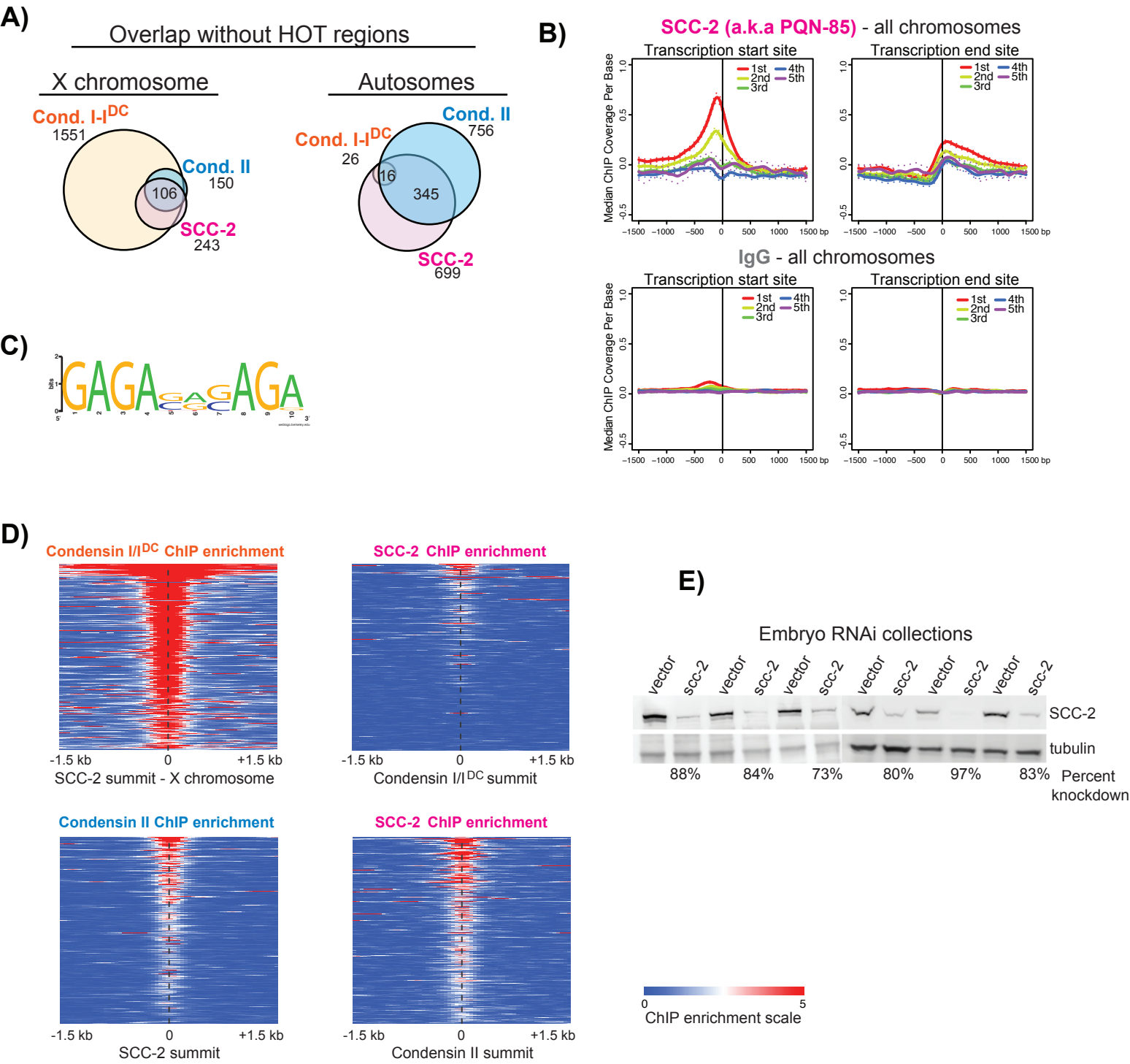

Supplement: Additional file 9: Figure S5 — (A) Overlap between binding sites of condensin I-IDC, condensin II and SCC-2 is shown. Numbers under each factor indicate the total number of binding sites. Overlapping numbers are based on the number of SCC-2 peaks. Those binding sites that overlap with HOT regions are removed from this analysis. (B) The median SCC-2 ChIP signal is plotted over the TSS and TES of all annotated genes. SCC-2 ChIP signal is enriched at promoters, and is proportional to transcription. Genes were ranked into five groups (highest expressed, 1st quintile; lowest expressed, 5th quintile) based on RNA level. As a control, IgG ChIP signal was also plotted across the transcription start and end sites (panels below). (C) Top 200 autosomal SCC-2 sites were used for motif search with MDScan [61]. The motif logo was created by web logo [64]. This motif is present in 58% of the SCC-2 peaks. (D) Heat maps demonstrating ChIP enrichment across binding peak summits for condensin or SCC-2. The peaks are ordered from strongest (top) to weaker binding (bottom). Condensin I-IDC ChIP signal is high across all SCC-2 peak summits on the X (top left panel). By contrast, SCC-2 ChIP signal is high for the subset of strongest condensin I-IDC binding sites, which include the majority of condensin IDC recruitment sites (top right panel). The heat map indicates that condensin II is enriched at SCC-2 sites (bottom left panel) and SCC-2 is enriched at condensin II sites (bottom right panel). (E) Western blot analysis of SCC-2 amount in embryos used for quantitative ChIP (qChIP) (Figure 5G). The percent reduction in SCC-2 amount was calculated based on tubulin loading control and the control RNAi. [file gb-2013-14-10-r112-S9.pdf]

Supplemental Figure 6

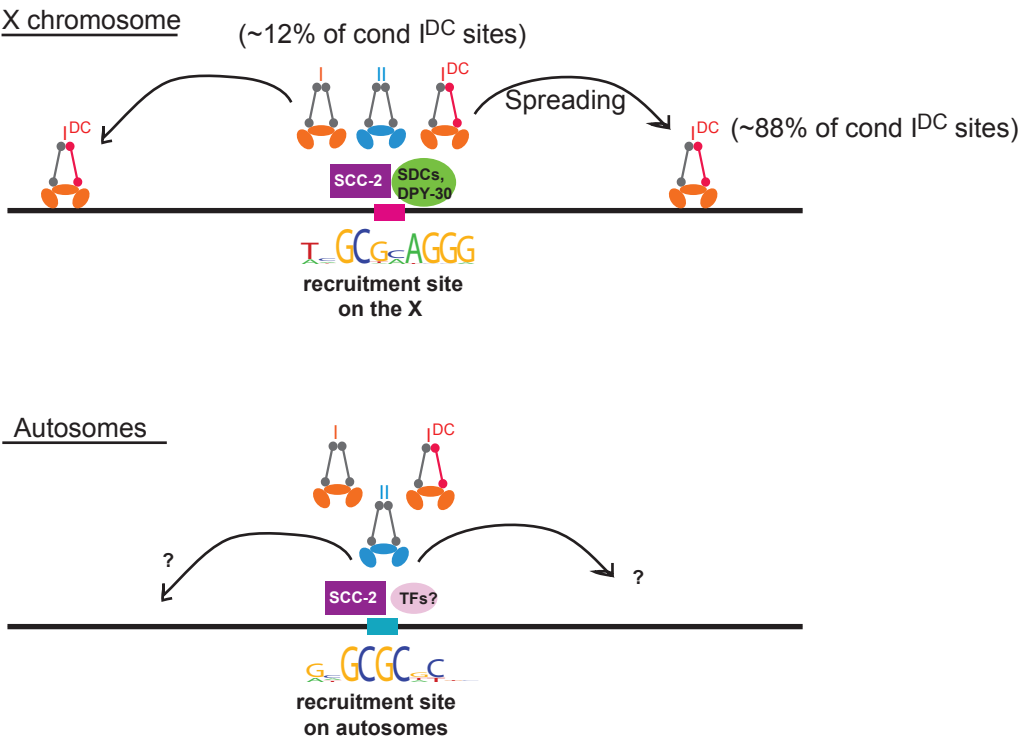

Supplement: Additional file 10: Figure S6 — Hypothetical model for condensin binding. Condensins are recruited to chromosomes at sites specified by DNA sequence motifs (colored boxes). These motifs may be recognized by recruiters and cofactors that can interact with condensins. For example, condensin IDC is able to interact with X-specific condensin recruiters, but not autosomal recruiters. There exists more than one condensin recruiter, since SDC-2 is required to bring condensin II to condensin IDC sites on the X chromosome but condensin II is independently recruited to autosomes. After recruitment, condensins spread onto nearby chromatin (indicated by arrows). For condensin IDC, at least 88% of the approximately 1,600 binding sites on the X are predicted to arise from spreading. The potential role of why SCC-2 is also recruited to the condensin IDC binding sites by SDC-2 is not clear. [file gb-2013-14-10-r112-S10.pdf]
