# Supplementary material for: Genome-wide analysis of condensin binding in Caenorhabditis elegans
Source: Genome Biol. 2013 Oct 14;14(10):R112. doi: 10.1186/gb-2013-14-10-r112 (PMC3983662; doi:10.1186/gb-2013-14-10-r112)
Supplement: Additional file 4: Figure S2 — (A) Enrichment or depletion of DPY-28 and HCP-6 binding sites at various genomic annotations are given. Random enrichment and p-values were calculated by a permutation test randomly distributing the condensin peaks 10,000 times. The data from individual subunits agrees with that shown for combined condensin I-IDC and condensin II sites in Figure 2A. (B) The overlap of condensin sites with various genomic annotations is shown separately for the X and autosomes. Note that the overlaps are not exclusive, and a peak can overlap with multiple annotations and vice versa. Genes represent all coding genes. Non-coding RNAs (ncRNAs) include all non-coding genes excluding tRNAs. Short and long classification is based on a 200 bp cutoff. Intergenic is defined as 1 kb away from any annotated gene, including coding and noncoding genes. 'Annotation coverage’ is the percentage of the genome covered with the annotation based on the criteria applied for overlap (for example, 1 kb promoter). [file gb-2013-14-10-r112-S4.pdf]

Supplemental Figure 2

A)

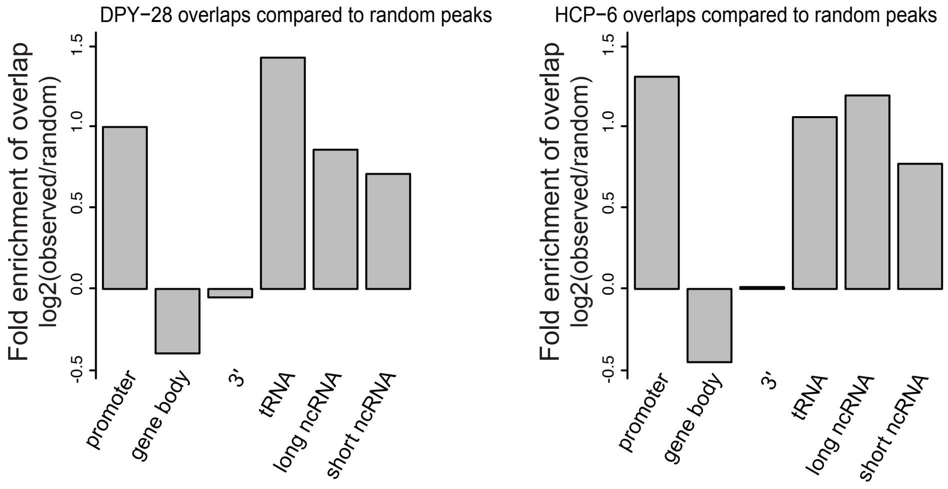

B)

	X chromosome: Percentage of Peaks*			on X	X chromosome: Percentage of Annotation*		
	I, I^{DC}	II	SCC-2	annotation coverage	I, I^{DC}	II	SCC-2
**1 kb promoter**	43	56	46	15	28	5	6
**gene body**	43	35	35	53	22	3	4
**1 kb down**	18	18	19	15	12	1	2
**intergenic**	14	15	16	19			
**short ncRNA**	26	27	32	16	39	5	7
**long ncRNA**	9	10	11	5.5	44	5	10
**tRNA**	8	10	13	2.4	58	11	20

	Autosomes: Percentage of Peaks*			on Aut	Autosomes: Percentage of Annotation*		
	I, I^{DC}	II	SCC-2	annotation coverage	I, I^{DC}	II	SCC-2
**1 kb promoter**	48	65	48	19	0.2	6	3
**gene body**	58	51	51	63	0.2	3	3
**1 kb down**	35	27	24	20	0.1	2	1
**intergenic**	0	10	13	13			
**short ncRNA**	0	18	22	8	0.6	6	7
**long ncRNA**	0	8	10	2.7	1.3	11	11
**tRNA**	8	2	4	0.7	1.2	6	13
